# Metabolic Reprogramming at the Maternal–Fetal Interface: Insights from Decidual Stromal Cells and Trophoblasts in Healthy Pregnancy Versus Recurrent Pregnancy Loss

**DOI:** 10.3390/ijms27146413

**Published:** 2026-07-19

**Authors:** Zhuo Liu, Zhuo Zhang, Yanjing Huang, Fan Li, Yuli Geng, Runan Hu, Mingmin Zhang, Yufan Song

**Affiliations:** 1 Institute of Integrated Traditional Chinese and Western Medicine, Tongji Hospital, Tongji Medical College, Huazhong University of Science and Technology, Wuhan 430030, China; d202482466@hust.edu.cn (Z.L.); zhuozhang26@163.com (Z.Z.); hyj13260684620@163.com (Y.H.); d202182041@hust.edu.cn (F.L.); 2 School of Clinical Medicine, Tsinghua Medicine, Tsinghua University, Beijing 100084, China; yuligeng@foxmail.com; 3 Department of Integrated Traditional Chinese and Western Medicine, Tongji Hospital, Tongji Medical College, Huazhong University of Science and Technology, Wuhan 430030, China; hurunan19970109@163.com

**Keywords:** metabolic reprogramming, decidual stromal cells, trophoblasts, recurrent pregnancy loss

## Abstract

Metabolic reprogramming at the maternal–fetal interface remodels enzymes, metabolites, and pathways to orchestrate decidualization, placentation, and immune tolerance, thereby sustaining cellular homeostasis and maternal-fetal adaptation. Disruption of this precisely coordinated metabolic reprogramming impairs these processes, contributing to recurrent pregnancy loss (RPL). This review focuses on the two most abundant cell types at the maternal–fetal interface: maternal-derived decidual stromal cells (DSCs) and embryo-derived trophoblasts, and systematically delineates their metabolic reprogramming in healthy pregnancy versus RPL. Recent advances reveal that altered glucose, lipid, amino acid, redox, and one-carbon metabolism in DSCs and trophoblasts drives defective decidualization, placentation, and immune tolerance, offering novel mechanistic insights into RPL etiology. These findings highlight potential metabolic biomarkers and therapeutic strategies, bridging mechanistic discoveries with translational opportunities for RPL.

## 1. Introduction

Recurrent pregnancy loss (RPL), defined as two or more consecutive pregnancy failures, affects 1–2% of couples and remains unexplained in over 50% of cases despite extensive clinical evaluation [1,2]. Immune dysregulation at the maternal–fetal interface has long been considered central, but this localized perspective may not fully capture the upstream drivers of RPL, particularly its recurrent nature [3,4].The maternal–fetal interface is composed of maternal-derived decidual stromal cells (DSCs), fetal-derived trophoblasts, and immune cells including decidual natural killer (dNK) cells, decidual macrophages (dMφ), and T-cells [5,6,7]. Their functional integration depends on precisely coordinated programs of differentiation, migration, and intercellular signaling, all of which are fundamentally driven by cellular metabolism [8,9].

Recent advances in immunometabolism have established that metabolic reprogramming is an active biological strategy that dictates cell fate and effector functions, rather than merely an adaptive response to energy demand [10,11,12,13]. At the maternal–fetal interface, DSCs undergo a Warburg-like glycolytic switch during decidualization, while trophoblasts remodel their metabolic programs to support proliferation, invasion, and syncytialization [14,15]. Metabolites such as lactate, succinate, and α-ketoglutarate have emerged as key signaling molecules that regulate gene expression through receptor-mediated pathways and epigenetic modifications, establishing a dynamic metabolic dialog within the interface [16,17,18]. High-throughput technologies, including single-cell RNA sequencing (scRNA-seq) and metabolomics, have further revealed marked metabolic heterogeneity and functional specialization at the interface, demonstrating that RPL-associated cellular dysfunction is frequently accompanied by profound metabolic perturbations [19,20,21,22].

Despite this progress, existing reviews have focused primarily on immune cell metabolism or on isolated stromal and trophoblast pathways, leaving the systematic integration of metabolic reprogramming across the two most abundant cell types at the interface largely unaddressed [10,23,24]. Therefore, this review establishes a comprehensive, dual-cell framework that delineates the metabolic adaptations of DSCs and trophoblasts in healthy pregnancy and their pathological dysregulation in RPL, offering novel insights into RPL etiology while identifying potential metabolic biomarkers and therapeutic strategies.

## 2. Metabolic Reprogramming of DSCs

Decidualization transforms endometrial stromal cells (EnSCs) into DSCs that support trophoblast invasion, vascular remodeling, and immune tolerance [20]. This transition activates glucose, amino acid, and sphingolipid metabolism, and its disruption contributes to RPL [14,20,25]. Metabolomic profiling of RPL decidua has identified 62 metabolites linked to pregnancy loss, underscoring the central role of metabolic dysregulation in RPL pathogenesis [19].

### 2.1. Glucose Metabolism Reprogramming

#### 2.1.1. Glucose Uptake Machinery

Glucose is the obligate energy substrate for decidualization and is acquired entirely through membrane bound transporters, primarily the glucose transporter (GLUT) family and sodium–glucose-linked transporter 1 (SGLT1) [26,27]. During the decidualization of human EnSCs, GLUT1 expression increases up to 10-fold through progesterone receptor (PR) signaling via insulin receptor substrate 2 (IRS2)-mediated mitogen-activated protein kinase (MAPK) and phosphatidylinositol 3-kinase (PI3K)/protein kinase B (AKT) pathways together with histone H3 lysine 27 (H3K27) acetylation-mediated epigenetic activation [28,29,30,31,32], while DSCs also secrete GLUT1-carrying extracellular vesicles that promote paracrine glucose uptake [33]. GLUT3 deficiency results in post implantation embryonic demise [34,35], GLUT4 undergoes insulin-stimulated translocation and its dysregulation contributes to decidualization defects in polycystic ovary syndrome [36], and GLUT8 is upregulated during decidualization and likely functions as an intracellular metabolite sensor [8,16]. SGLT1, the sole high-affinity Na^+^-coupled transporter in the endometrium drives glycogen accumulation even under glucose limitation [37], and its marked reduction in RPL mid-luteal endometrium cautions against periconceptional SGLT1 inhibitor use [38,39]. Functional studies in primary human EnSCs demonstrated that glucose scarcity constrains impair decidualization, at least in part, by suppressing forkhead box protein O1 (FOXO1) and reducing H3K27 acetylation at the promoters of prolactin (PRL) and insulin-like growth factor binding protein 1 (IGFBP1) [40,41]. In the context of maternal metabolic disorders, endometrial GLUT1 expression is paradoxically suppressed despite systemic hyperinsulinemia, thereby limiting glucose availability to DSCs and increasing susceptibility to RPL [42,43], whereas metformin has been shown to mitigate miscarriage risk by restoring GLUT expression [44,45]. Notably, the observation of compensatory GLUT1 upregulation in decidual tissue from RPL patients suggests that tightly regulated GLUT1 levels, rather than maximal expression, are required to maintain decidual integrity [14].

#### 2.1.2. Glucose Metabolic Pathway

In vivo and in vitro studies in humans and mice demonstrate that decidualization induces a Warburg-like glycolytic shift, which, unlike the oxygen insensitive Warburg effect in cancer, is driven by the physiological hypoxia of early pregnancy [26]. The shift is mediated by coordinated upregulation of hexokinase 2 (HK2), lactate dehydrogenase A (LDHA), phosphofructokinase 1 (PFK1), pyruvate kinase M2 (PKM2), pyruvate dehydrogenase kinase, and hypoxia inducible factor 1α (HIF-1α) [26,46,47]. It accelerates pyruvate to lactate conversion and lactate shuttling via monocarboxylate transporter 4 (MCT4) and monocarboxylate transporter 1 (MCT1) to meet acute energy demands and generate an acidic microenvironment that supports cell proliferation and uterine remodeling [46,48].

The hexosamine biosynthesis pathway (HBP), a glycolytic branch, produces uridine diphosphate N-acetylglucosamine (UDP-GlcNAc) for O-GlcNAcylation, linking metabolic status to cellular function [49]. In decidualizing human endometrium, O-linked β-N-acetylglucosamine transferase (OGT) and global O-linked N-acetylglucosamine (O-GlcNAc) levels decline whereas endoplasmic reticulum-specific EGF domain-containing O-GlcNAc transferase is upregulated, contributing to metabolic reprogramming via the decidualization gene network and the adropin encoding gene *ENHO* [50]. Under metabolic stress conditions such as maternal obesity and gestational diabetes mellitus, excessive HBP activation elevates UDP-GlcNAc levels and O-GlcNAcylation, which disrupts insulin signaling and impairs EnSC decidualization, thereby increasing RPL susceptibility [49,50].

In both humans and mice, decidualization activates the pentose phosphate pathway (PPP) and its rate-limiting enzyme glucose-6-phosphate dehydrogenase (G6PD) [46,51,52]. The metabolic sensor G protein-coupled receptor 120 potentiates this shift by enhancing GLUT1-mediated glucose uptake and G6PD-driven PPP flux, and its downregulation is associated with increased abortion susceptibility [25,52]. The tricarboxylic acid (TCA) cycle is partially restrained during decidualization of human EnSCs, with mild isocitrate dehydrogenase 2 downregulation but elevated succinate dehydrogenase A and fumarate hydratase. Nevertheless, TCA intermediates remain essential for amino acid and fatty acid synthesis [26,47,53].

scRNA-seq of decidual tissue from RPL patients and healthy controls identified three metabolically distinct DSC subsets, including myofibroblastic DSCs, which rely on oxidative phosphorylation (OXPHOS), the TCA cycle, and the pentose phosphate pathway, and inflammatory and glycolytic DSCs, which exhibit globally suppressed metabolism with reinforced glycolysis and hypoxic features [14]. In RPL, persistent pathological hypoxia drives expansion of inflammatory DSCs and glycolytic DSCs with concomitant upregulation of HIF-1α, LDHA, and HK2, indicating that dysregulated glycolytic programming and compromised metabolic flexibility contribute to decidual dysfunction and pregnancy loss [14,54,55].

#### 2.1.3. Glucose Metabolites

During decidualization, glucose metabolism is rewired to favor glycolytic intermediates that act as signaling molecules, epigenetic modifiers, and immunomodulators [46]. Fructose-1,6-bisphosphate (FBP) has emerged as a central immune-metabolic checkpoint. Its accumulation depends on the PFK1/FBP1 switch [56], and DSCs upregulate PFK1 and downregulate FBP1 relative to EnSCs, promoting FBP synthesis and secretion [56]. FBP binds PKM2 to form a positive feedback loop that reinforces FBP accumulation and ATP generation [56,57]. Beyond its bioenergetic role, FBP stimulates interleukin-27 (IL-27) secretion via extracellular signal-regulated kinase (ERK)/FBJ murine osteosarcoma viral oncogene homolog signaling, thereby driving M2-like (anti-inflammatory) macrophage polarization that facilitates trophoblast invasion and maternal-fetal immune tolerance [56,58,59]. In women with unexplained recurrent pregnancy loss (URPL), reduced FBP in plasma and decidua correlates with deficient decidual IL-27 and M2-like macrophages. Exogenous FBP restores macrophage polarization and prevents embryo loss in murine models, establishing proof-of-concept for metabolic intervention in miscarriage [8,56,60].

Additionally, glycolysis-derived acetyl-coenzyme A (acetyl-CoA) not only serves as a substrate for the TCA cycle but also participates in histone acetylation to regulate gene expression [47,61]. PPP-derived nicotinamide adenine dinucleotide phosphate sustains antioxidant capacity, while ribose-5-phosphate supports the nucleotide synthesis required for proliferating stromal cells [26,52].

Human and mouse studies, both in vivo and in vitro, demonstrate that lactate levels rise following decidualization [17,46]. As a substrate for lactylation, lactate mediates multiple epigenetic regulations. Histone H4 lysine 12 lactylation enhances HIF-1α expression, forming a positive feedback loop that drives decidualization [61], whereas histone H3 lysine 18 lactylation (H3K18la) modulates redox homeostasis and apoptosis to facilitate endometrial remodeling [62]. In URPL, transcriptomic and metabolomic profiling of primary stromal cells uncovered a pathogenic HK2–H3K18la–cut-like homeobox 1 (CUX1)–senescence-associated secretory phenotype (SASP) axis. HK2-driven glycolysis elevates H3K18la at the CUX1 promoter, upregulating SASP factors that drive stromal senescence and impair decidualization. Glycolysis inhibition or CUX1 depletion reversed these defects in vitro, and CUX1 knockdown in a URPL mouse model further validated the rescue of decidualization, offering a translatable metabolic strategy [55,56].

### 2.2. Lipid Metabolism Reprogramming

Lipid metabolism governs energy homeostasis and signal transduction at the maternal–fetal interface [63]. scRNA-seq of the human maternal–fetal interface reveals that DSCs exhibit the highest lipid metabolic activity among all cell types, with highly mature DSCs preferentially activating sphingolipid metabolism [54]. This sphingolipid-enriched reprogramming is evolutionarily conserved, as similar metabolic shifts occur during early pregnancy in pigs, cattle, and mice [54].

#### 2.2.1. Fatty Acid Metabolism

Efficient lipid uptake is required for decidualization. Wilms tumor 1 upregulates very low-density lipoprotein receptor transcription to promote lipoprotein uptake and lipid droplet accumulation [64], alongside high expression of fatty acid-binding protein 4 [65]. This process coincides with elevated saturated fatty acid levels and reduced levels of polyunsaturated fatty acids (PUFAs) and sterols [66], yet excessive SFA exposure suppresses decidualization by impairing mitochondrial function [67,68]. In RPL, reduced fatty acid-binding protein 5 in DSCs represses mitochondrial ribosomal protein L17, disrupting OXPHOS, and triggering intrinsic apoptosis [69].

In both humans and mice, decidualization coordinately activates lipogenic and lipolytic programs in DSCs, with fatty acid oxidation (FAO) being indispensable [47,70]. pregulated long chain acyl CoA synthetase 1/4 (ACSL1/4) promotes fatty acid activation and sustains FAO, while also regulating the PI3K/AKT/FOXO1 axis to upregulate decidual markers and mitigate high lipid mediated oxidative stress and mitochondrial injury [68,71]. During human ESC decidualization, upregulation of carnitine palmitoyltransferase 1 and 2 (CPT1/2) enhances β-oxidation of long- and medium-chain fatty acids. Metabolomic profiling revealed that octanoic acid production increased upon decidualization, facilitating embryo implantation [72]. In decidua from spontaneous abortion patients, CPT1 protein is markedly downregulated. In parallel, metabolomic profiling of RPL decidua reveals a broad decline in free carnitine and acylcarnitines of all chain lengths, suggesting impaired FAO and energy deficiency in DSCs [19,63]. In primary human and mouse EnSCs, CPT1 deficiency or FAO blockade disrupts decidualization via PI3K/AKT-mediated FOXO1 degradation, and uterine CPT1 knockdown in mice recapitulates these defects and triggers embryo loss [51,71,73]. Together, these findings identify CPT1 and acylcarnitines as potential diagnostic biomarkers and therapeutic targets.

**Figure 1:**
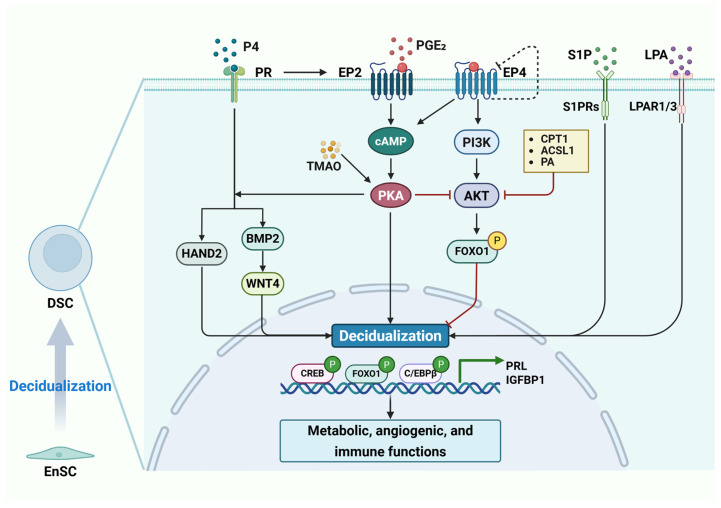
Core lipid signals regulating human EnSC decidualization into DSC. PGE_2_ binding to EP2 activates the cAMP-PKA pathway, which phosphorylates transcription factors including CREB, FOXO1, and C/EBPβ to initiate differentiation. PKA also enhances PR activity and cooperates with the PR-HAND2/BMP2-WNT4 axis to drive the decidual gene network, marked by PRL and IGFBP1. Concurrently, PKA facilitates AKT inactivation to promote chromatin remodeling. The EP4-PI3K-AKT cascade suppresses decidualization (red lines). TMAO promotes PKA signaling and further supports decidualization. Lipid mediators S1P and LPA promote decidualization via S1PRs and LPAR1/3. CPT1, ACSL1, and PA modulate AKT activity to balance differentiation. Abbreviations: BMP2, bone morphogenetic protein 2; cAMP, cyclic adenosine monophosphate; C/EBPβ, CCAAT/enhancer-binding protein beta; HAND2, heart and neural crest derivatives expressed 2; PA, phosphatidic acid; S1PRs, sphingosine-1-phosphate receptors; TMAO, trimethylamine N-oxide; WNT4, Wnt family member 4. Created in BioRender. Runan, H. (2026) https://BioRender.com/hhfzqhf (accessed on 12 July 2026).

Prostaglandins (PGs) derived from arachidonic acid (ARA) are mediators of decidualization, implantation, and immune tolerance at the maternal–fetal interface [74,75]. ARA is released from membrane phospholipids by phospholipase A2 (PLA2) and converted by cyclooxygenase (COX) into bioactive products including prostaglandin E_2_ (PGE_2_) and prostaglandin F_2_α [76]. In human EnSCs, PGE_2_ acts as a progesterone (P4)-synergistic decidualization signal that activates protein kinase A (PKA) via prostaglandin E receptor 2 (EP2) while EP4 restrains AKT [77,78,79]. Dominant PKA signaling directly phosphorylates transcription factors including cAMP response element-binding protein (CREB), FOXO1, and CCAAT/enhancer-binding protein β to initiate early differentiation. It also enhances PR activity and cooperates with the PR–heart and neural crest derivatives expressed 2 and bone morphogenetic protein 2–Wnt family member 4 pathway [76,80]. Moreover, PKA promotes AKT inactivation via downregulation of cancerous inhibitor of protein phosphatase 2A, facilitating chromatin remodeling [81]. This sequence drives the core decidualization gene network marked by PRL and IGFBP1, ultimately orchestrating cellular morphological and functional remodeling [78,82] (Figure 1).

In RPL, hyperactive ARA metabolism driven by upregulated PLA_2_ isozymes and COX-2 in DSCs leads to excessive PGs accumulation, impairing DSC function and triggering local inflammation [83,84,85]. Notably, DSC-derived PRL can regulate the ARA metabolic pathway in an autocrine and paracrine manner. PRL pretreatment reduces embryo loss in a mouse model of ARA-associated miscarriage and improves the maternal fetal immune homeostasis, suggesting that PRL may act as a promising therapeutic target for reducing miscarriage risk [84].

#### 2.2.2. Phospholipid Metabolism

Phospholipid remodeling during peri implantation provides essential substrates for decidualization. In women, endometrial fluid glycerophospholipids and PUFAs are markedly altered between implantative and non-implantative in vitro fertilization cycles [74]. Phosphatidic acid (PA) promotes decidualization in human EnSCs by facilitating AKT-protein phosphatase 2A interaction to attenuate FOXO1 phosphorylation [86]. Lysophosphatidic acid (LPA), generated from lysophosphatidylcholine (LPC) by autotaxin (ATX) signals via lysophosphatidic acid receptors (LPARs) to regulate decidualization and implantation [87,88]. During decidualization of human EnSCs, ATX, LPAR1, and lipid phosphate phosphatases (LPP1/3) are upregulated, highlighting the critical role of LPA metabolism in decidualization and embryo implantation [88,89].

In RPL, widespread glycerophospholipid dysregulation is observed in the decidua, with downregulation of PA, phosphatidylserine, phosphatidylglycerol, phosphatidylcholine, phosphatidylethanolamine, and lysophospholipids, alongside reduced phospholipase A2 group IIE (PLA2G2E) expression, indicating concurrent defects in glycerophospholipid synthesis and degradation. These alterations compromise the structural integrity and signaling function of DSCs, potentially triggering excessive inflammation and constituting a critical metabolic basis for RPL (Figure S1A,C) [19].

#### 2.2.3. Sphingolipid Metabolism

Single-cell transcriptomics of the human maternal–fetal interface reveals that sphingolipid metabolism is markedly upregulated during decidualization, with DSC subsets expressing high decidual markers exhibiting the strongest sphingolipid activity [54]. These cells secrete sphingosine-1-phosphate (S1P) to villous cytotrophoblasts, mediating maternal–fetal crosstalk. S1P engages sphingosine 1 phosphate receptors to activate downstream signaling that boosts decidualization and COX-2 expression, and through crosstalk with PG signaling, orchestrates immune tolerance, angiogenesis, and trophoblast phenotypes [54,70]. In human EnSCs, decidualization upregulates sphingosine kinase 1 (SPHK1), sphingosine-1-phosphate phosphatase 1, and sphingosine-1-phosphate lyase 1, ensuring high S1P turnover for precise spatiotemporal control [89]. This sphingolipid activation is conserved in early-pregnancy mice [8,74]. Conversely, in *Sphk*-deficient mice, decidualization defects, vascular instability, and excessive neutrophil extracellular trap (NET) formation at the maternal–fetal interface trigger embryonic loss [90]. NETs are also induced in human neutrophils exposed to *Sphk*-deficient decidual cells, and pharmacological NET inhibition alleviates vascular damage and pregnancy loss in these mice, confirming NETs as a pathogenic driver in URPL [91].

In RPL patients, decidual sphingolipid metabolism shifts toward sphingomyelin synthesis. Specific sphingomyelin and ceramide subtypes are markedly elevated, accompanied by upregulated sphingomyelin synthase 1 and unchanged sphingomyelin phosphodiesterase 1/3, indicating that sphingomyelin accumulation arises from enhanced synthesis rather than attenuated degradation. This sphingomyelin overload disrupts decidualization, immune regulation, and uteroplacental vascular function, resulting in compromised embryonic development (Figure S1B,C) [19].

#### 2.2.4. Trimethylamine N-Oxide (TMAO) Metabolism

Non-targeted NMR-based metabolomic profiling of decidual tissues from RPL patients versus normal controls revealed a significant depletion of TMAO [92]. DSCs autonomously synthesize TMAO from choline via flavin-containing monooxygenase 3 (FMO3), which is upregulated by the cAMP-PKA-CREB1 axis during decidualization [92]. TMAO binds 14-3-3η to enhance its interaction with 3-phosphoinositide-dependent protein kinase 1 (PDK1), thereby relieving PDK1-mediated suppression of FOXO1 and promoting its nuclear translocation and decidual gene transcription. In RPL, disrupted FMO3 expression leads to TMAO deficiency and FOXO1 hyperphosphorylation. In mice, FMO3 inhibition or dietary choline restriction lowers uterine TMAO, impairs decidualization, and causes embryo resorption. Notably, exogenous TMAO restores decidualization in approximately 15% of primary DSCs from RPL patients, identifying a subset amenable to metabolic intervention and positioning this pathway as a therapeutic target [92].

### 2.3. Amino Acid Metabolic Reprogramming

Amino acid uptake into DSCs is mediated by amino acid transporters of the solute carrier superfamily [16]. Integrative scRNA-seq datasets reveal a global reprogramming of amino acid metabolism during the EnSC-to-DSC transition, with early-stage cells displaying high amino acid metabolic activity that suggests a critical role in decidual initiation and phenotype maintenance [54]. The reprogramming of alanine, aspartate, glutamate, and glutathione metabolism is highly conserved across placental mammals, indicating that amino acid metabolic reprogramming is an evolutionarily conserved mechanism for sustaining pregnancy [54].

#### 2.3.1. Glutamine Metabolism

Glutamine metabolism is conspicuously upregulated in decidualizing EnSCs [54]. Glutaminase 1 (GLS1) converts glutamine to glutamate, while glutamine synthetase (GS) regenerates glutamine from glutamate, forming a substrate cycle that maintains glutamine homeostasis. Glutamate has two major metabolic fates. First, transaminases or glutamate dehydrogenase convert glutamate to α-ketoglutarate (α-KG), fueling the TCA cycle and regulating decidualization gene expression, including PRL and IGFBP1, through H3K27 demethylation [93]. Second, glutamate drives glutathione (GSH) synthesis via glutamate-cysteine ligase, and GSH metabolic activity increases progressively with EnSC maturation, protecting DSCs from oxidative injury [8,54]. The glutamate derivative N-acetylaspartylglutamate exerts anti-inflammatory effects and is downregulated in RPL decidua, potentially contributing to exaggerated local inflammation [19].

Multiple studies of RPL decidua reveal disruption of both glutamine metabolic branches. Impaired glutamine–glutamate-α-KG flux compromises decidualization via aberrant histone methylation and energy deficit [93]. Concurrently, GSH metabolic deficiency and local iron overload further downregulate GSH and glutathione peroxidase 4 (GPX4), thereby inducing ferroptosis in DSCs [19,94]. Supplementation with α-KG, GSH, or its precursor cysteine effectively rectifies these defects in mouse models, representing a putative strategy to restore normal decidualization in RPL patients [93,94].

Notably, scRNA-seq-based metabolic crosstalk predictions reveal that a decidual subpopulation with elevated alanine–aspartate–glutamate metabolism secretes glutamine to syncytiotrophoblasts (STBs), where it activates the mechanistic target of rapamycin (mTOR) pathway to foster placental protein synthesis and proliferation [54]. These findings indicate that DSCs direct maternal–fetal communication through program-specific metabolite secretion, and dysregulation of this metabolic dialog may exacerbate pregnancy loss in RPL.

#### 2.3.2. Arginine Metabolism

scRNA-seq datasets of humans reveal that arginine–proline metabolism is activated during early decidualization and shapes cellular behaviors throughout differentiation [54]. DSCs maintain arginine supply via de novo synthesis through argininosuccinate synthase 1, driven by cAMP/PKA/CREB signaling [8]. Arginine is converted by arginase to ornithine, which is either used for proline synthesis or decarboxylated by ornithine decarboxylase (ODC) to initiate polyamine synthesis, supporting cell proliferation, differentiation, and angiogenesis [95]. Arginine also serves as a substrate for nitric oxide synthase (NOS) to produce nitric oxide (NO), a pivotal regulator of uterine vasodilation [96], and dietary arginine facilitates implantation in rats via the PI3K/mTOR/NO pathway [97]. Proline supplementation rescues human EnSC decidualization under nutritional stress in vitro, underscoring the arginine–proline axis [98].

In RPL, DSCs present pronounced arginine metabolic disturbances, including L-citrulline accumulation, impaired arginine-to-NO conversion, and potentially compromised polyamine and proline synthesis [19,98]. These defects disrupt decidualization, reduce decidual perfusion, and weaken DSC-mediated immunosuppression of dNK and T-cells, thereby breaking maternal–fetal immune tolerance and driving pregnancy failure [19].

#### 2.3.3. Tryptophan Metabolism

In human EnSCs, tryptophan metabolism regulates decidualization through two opposing branches. Physiologically, indoleamine 2,3 dioxygenase 1 (IDO1) converts tryptophan to kynurenine, which activates aryl hydrocarbon receptor (AHR) signaling to promote decidualization [99]. Concurrently, monoamine oxidase A (MAOA) and MAOB are upregulated to clear serotonin, preventing its local accumulation. In women with recurrent implantation failure, a condition also characterized by decidualization impairment, downregulated MAOA and MAOB lead to serotonin overload, which disrupts decidualization by impairing phosphatidylcholine metabolism, a defect that can be rescued by phosphatidylcholine supplementation in vitro [100]. In mice, a tryptophan free diet severely impairs decidualization and implantation despite elevated IDO and AHR levels, indicating that tryptophan is indispensable for early pregnancy beyond its role as a kynurenine precursor [101]. Conversely, excessive tryptophan is also detrimental. In the Rotterdam periconceptional cohort, elevated maternal kynurenine was associated with impaired uteroplacental vascular development, and increased serotonin linked to hypertension and preeclampsia risk [102]. Thus, a balanced tryptophan metabolic flux is essential for successful pregnancy, as deviations in either direction predispose to adverse outcomes.

#### 2.3.4. Branched-Chain Amino Acid (BCAA) Metabolism

Senescent DSCs accumulate physiologically during decidualization, with their homeostasis maintained by dNK cells [103]. These cells upregulate solute carrier family 3 member 2 (SLC3A2), enhancing BCAA uptake [104,105]. In RPL, the senescent DSC population expands alongside elevated SLC3A2 and BCAA influx, and leucine drives DSC senescence via p38 MAPK [106]. In vitro, leucine deprivation limits senescence, whereas a high leucine diet exacerbates DSC senescence and precipitates miscarriage in mice [106]. Metformin protects against abortion in D galactose-induced senescent models [106], highlighting the therapeutic promise of targeting DSC senescence homeostasis in RPL [104,105].

#### 2.3.5. Cysteine and Methionine Metabolism

Cysteine and methionine metabolism acts as a core amino acid pathway activated in early decidualization, with peak activity in immature DSC subsets [54]. This pathway couples GSH synthesis and methyl donor metabolism, thereby preserving redox homeostasis and epigenetic stability. Related genes are upregulated upon decidual initiation and decline with differentiation, indicating a primary role in early stress adaptation and proliferation control [54]. In RPL, defective vitamin digestion and absorption, particularly vitamin B6 dysregulation in DSCs, may disrupt methionine–cysteine flux, remotely compromising decidual integrity and function [19].

**Figure 2:**
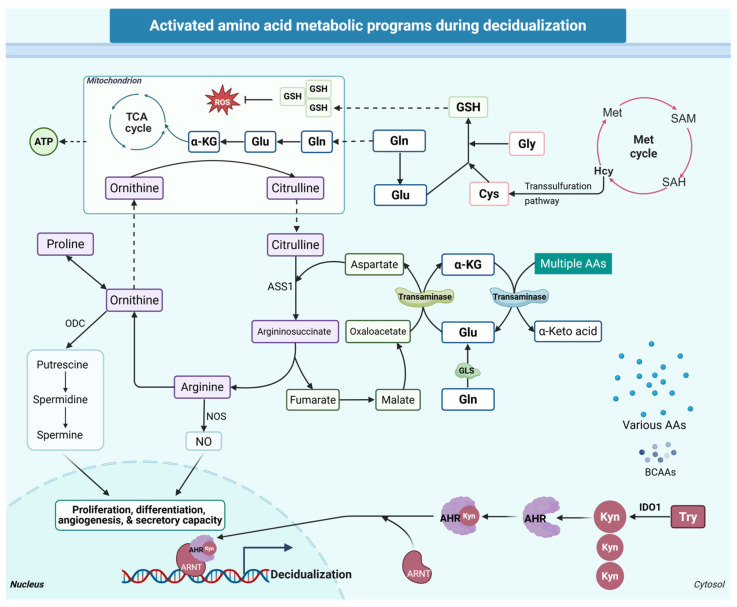
Amino acid metabolic reprogramming during human decidualization. The metabolic homeostasis of multiple amino acids, including BCAAs, is indispensable for DSC cellular functions. Among them, glutamine, arginine, and tryptophan metabolism constitute the most pivotal branches. GLS converts Gln to Glu, which enters the TCA cycle as α-KG to sustain ATP synthesis and fuel GSH production for ROS elimination. Glu, Gly, and Cys jointly synthesize GSH via the transsulfuration pathway supported by the Met cycle. In arginine metabolism, ODC converts ornithine to polyamines, while ASS1 converts citrulline to argininosuccinate and subsequently to arginine. NOS then catalyzes arginine to produce NO. The malate–fumarate shuttle links arginine synthesis to the mitochondrial TCA cycle. Polyamines and NO jointly regulate DSC proliferation, differentiation, angiogenesis, and secretory function. In tryptophan metabolism, IDO1 catabolizes Trp to Kyn, which binds AHR. The AHR-ARNT complex then translocates to the nucleus to transcriptionally promote decidualization. Abbreviations: AAs, amino acids; ARNT, aryl hydrocarbon receptor nuclear translocator; ASS1, argininosuccinate synthase 1; Cys, cysteine; Gln, glutamine; Glu, glutamate; GLS, glutaminase; Gly, glycine; Hcy, homocysteine; Kyn, kynurenine; Met, methionine; ODC, ornithine decarboxylase; SAH, S-adenosylhomocysteine; SAM, S-adenosylmethionine; Try, tryptophan. Created in BioRender. Runan, H. (2026) https://BioRender.com/rcwdw6d (accessed on 8 July 2026).

Collectively, DSCs rewire amino acid metabolism during decidualization to sustain biosynthesis, energy, and redox balance. Glutamine fuels the TCA cycle and GSH synthesis, the methionine–cysteine axis drives one-carbon and GSH metabolism, and arginine supplies polyamines and NO. BCAA and tryptophan catabolism further support proliferation, differentiation, angiogenesis, and secretion (Figure 2).

### 2.4. Metabolic Network Integration in DSCs

**Figure 3:**
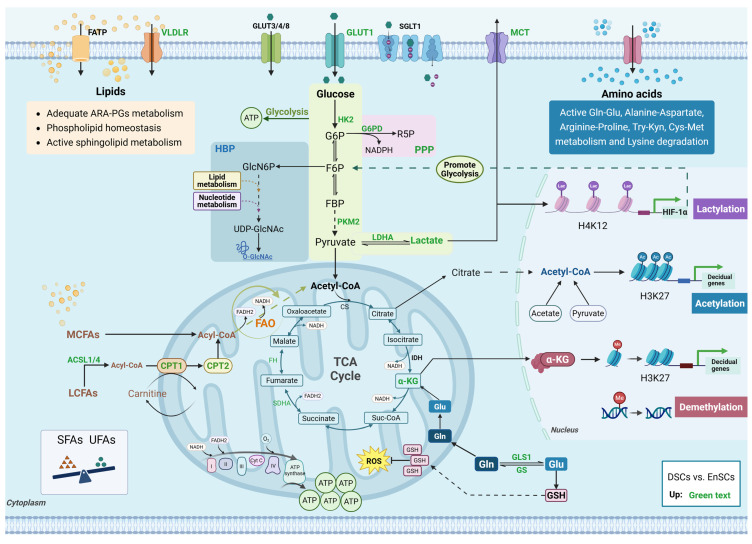
Schematic of metabolic reprogramming in DSCs during healthy decidualization. Decidualization of EnSCs into DSCs involves extensive metabolic rewiring. Glycolysis and the PPP are activated. Lipid metabolism maintains membrane integrity, metabolic homeostasis, and signaling. Amino acid metabolism reinforces one-carbon flux and antioxidant capacity. The coordinated crosstalk among these networks supplies energy, biosynthetic precursors, and regulatory signals that collectively drive DSC secretory function and cell fate commitment. Green labeled pathways and molecules are significantly upregulated in DSCs relative to EnSCs. Abbreviations: Acyl CoA, acyl coenzyme A; CS, citrate synthase; F6P, fructose 6-phosphate; FADH_2_, flavin adenine dinucleotide reduced form; FATP, fatty acid transport protein; FH, fumarate hydratase; G6P, glucose 6-phosphate; GlcN6P, glucosamine 6-phosphate; LCFAs, long-chain fatty acids; MCFAs, medium-chain fatty acids; NADH, reduced nicotinamide adenine dinucleotide; R5P, ribose 5-phosphate; ROS, reactive oxygen species; SFAs, saturated fatty acids; Suc-CoA, succinyl–coenzyme A; UFAs, unsaturated fatty acids; VLDLR, very low-density lipoprotein receptor. Created in BioRender. Runan, H. (2026) https://BioRender.com/t6fkhxi (accessed on 8 July 2026).

**Figure 4:**
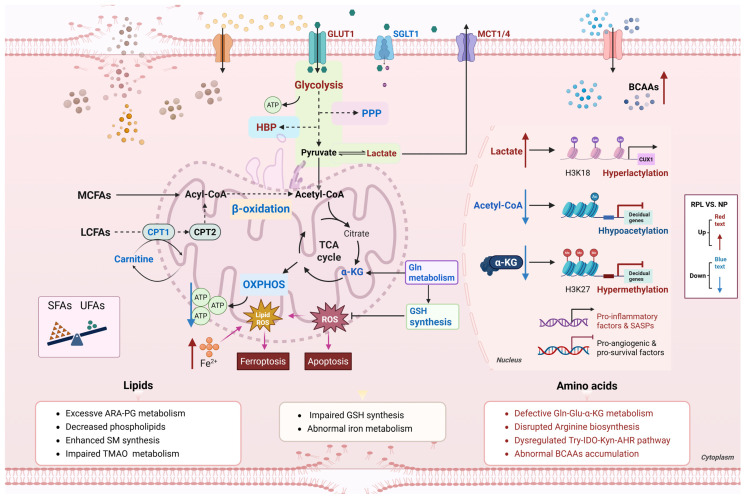
Aberrant metabolic rewiring in DSCs from RPL. Compared with normal pregnancy (NP), DSCs from patients with RPL exhibit profound metabolic dysregulation. Glycolysis is excessively activated, leading to enhanced lactate production and histone hyperlactylation. FAO and phospholipid synthesis are suppressed, whereas ARA-PG metabolism and sphingomyelin synthesis are exaggerated. Amino acid metabolism is disrupted at multiple nodes, including impaired Gln-Glu-α-KG flux, defective arginine biosynthesis, and dysregulated Try-Kyn-AHR signaling, together with abnormal BCAAs accumulation. Collectively, these metabolic disturbances induce energy insufficiency, membrane damage and signaling disorder, impairing DSC function, initiating apoptosis and ferroptosis, and further breaking maternal–fetal interface homeostasis. Red-labeled molecules and upward arrows indicate upregulation in DSCs from RPL relative to NP, while blue-labeled molecules and downward arrows indicate downregulation. Created in BioRender. Runan, H. (2026) https://BioRender.com/o7i6qur (accessed on 8 July 2026).

The metabolic pathways described above, including glycolysis, FAO, amino acid metabolism, and sphingolipid turnover, operate as an integrated network rather than in isolation [32,47,54]. Key metabolites, including acetyl-CoA, α-KG, lactate, and FBP, serve dual roles as metabolic intermediates and signaling molecules, coordinating DSC bioenergetics with epigenetic regulation and paracrine communication [17,41,56] (Figure 3). In RPL, disruption at any node propagates through this network, producing the triad of bioenergetic failure, membrane compromise, and epigenetic dysregulation that underlies decidualization defects [14,19] (Figure 4). The metabolic dysfunction of DSCs extends beyond cell autonomous effects to disrupt the broader decidual microenvironment, as discussed in Section 4.1.

## 3. Trophoblast Metabolic Reprogramming

Trophoblasts serve as the functional core of the maternal–fetal interface, orchestrating embryo implantation, placental morphogenesis, maternal–fetal nutrient transport, and immune tolerance [107]. Programmed metabolic reprogramming represents a spatiotemporally regulated feature of trophoblast development, enabling precise control over lineage specification, proliferation, invasion, and differentiation through remodeling of metabolic pathways and their crosstalk with signaling networks [9,15,21]. Disruption of this finely tuned metabolic circuitry directly compromises trophoblast function, impairs placental development, and destabilizes maternal–fetal interface homeostasis, thereby driving the pathogenesis of RPL [108,109,110,111]. This chapter systematically dissects the key molecular mechanisms underlying trophoblast metabolic reprogramming across three major metabolic pathways and their pathological relevance to RPL.

### 3.1. Glucose Metabolic Reprogramming

#### 3.1.1. Glucose Transport

The GLUT family exhibits cell-subtype-specific expression in human trophoblasts [53]. GLUT1, constitutively expressed in cytotrophoblasts (CTBs) and STBs, is the primary glucose transporter [53,112]. GLUT3 is enriched in proliferative trophoblast stem cells (TSCs) and CTBs, sustaining glycolytic flux via high-affinity glucose uptake [113], while insulin-inducible GLUT4 in STBs enables regulated fetal glucose supply [35,114]. In human HTR-8/SVneo trophoblasts, *SLC2A1* (encoding GLUT1) deletion shifts metabolism from glycolysis to OXPHOS, triggering mitochondrial calcium overload, reactive oxygen species overproduction, and endoplasmic reticulum stress, which together impair PI3K/AKT signaling, reduce migration and invasion, and disrupt epithelial–mesenchymal transition (EMT) [115,116].

#### 3.1.2. Developmental Metabolic Reprogramming

**Figure 5:**
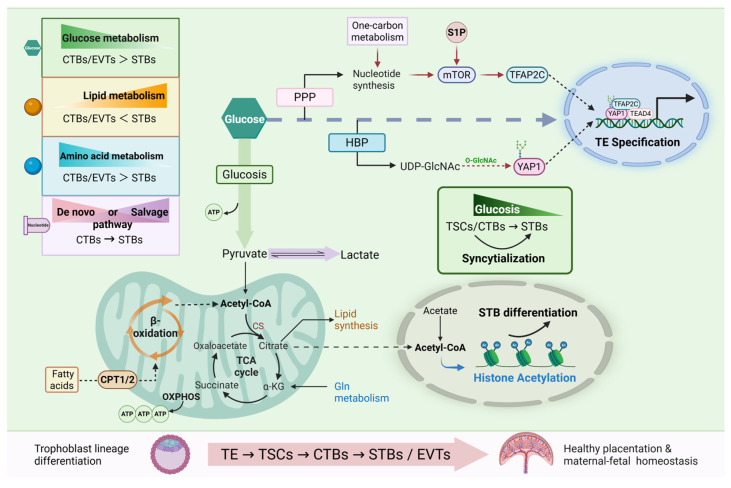
Developmental metabolic reprogramming during human trophoblast lineage differentiation. Metabolic programming couples metabolic state with differentiation cues to determine trophoblast lineage identity. In blastocyst TE, glucose flux through the HBP drives YAP1 nuclear translocation, while the PPP cooperates with S1P-mTOR signaling to promote assembly of the YAP1-TEAD4-TFAP2C complex, specifying TE identity. TE sequentially differentiates into TSCs, CTBs, and terminally to STBs and EVTs. During CTB syncytialization, glycolysis declines and residual carbon is redirected to acetyl-CoA for histone acetylation, activating syncytialization genes and shifting metabolism toward lipid metabolism and FAO to support STB endocrine and nutrient exchange functions. Trophoblast subtypes display distinct metabolic signatures, with CTBs and EVTs exhibiting higher glucose and amino acid metabolism and lower lipid metabolism relative to STBs, while nucleotide metabolism is remodeled throughout the CTB-to-STB transition. Abbreviations: TEAD4, TEA domain transcription factor 4; TFAP2C, transcription factor AP-2 gamma. Created in BioRender. Runan, H. (2026) https://BioRender.com/ixo77n6 (accessed on 8 July 2026).

Developmental metabolic programming coordinates metabolic state with differentiation cues to determine trophoblast lineage fate [15,21]. In the trophectoderm (TE) of preimplantation blastocysts, glucose flux through the HBP drives Yes-associated protein 1 (YAP1) nuclear translocation, while the PPP couples with S1P signaling to activate mTOR, promoting assembly of the YAP1–TEA domain transcription factor 4–transcription factor AP-2 gamma complex that specifies TE lineage identity [21]. The TE gives rise to TSCs, which progress to CTBs and ultimately give rise to STBs and extravillous trophoblasts (EVTs). In the first-trimester human placenta, TSCs and CTBs rely on aerobic glycolysis driven by PI3K/AKT/mTOR signaling to sustain stemness, proliferation, and invasion [15]. During syncytialization, glycolytic flux declines and residual carbon is redirected to acetyl CoA production for histone acetylation, which activates syncytialization genes and drives a metabolic shift toward lipid metabolism and FAO to support endocrine function and nutrient exchange [15] (Figure 5). In vitro, glycolytic insufficiency redirects TSCs toward a pro-inflammatory fate, which can be rescued by exogenous acetate through replenishment of acetyl-CoA, highlighting a potential strategy for correcting trophoblast metabolic disturbances [15]. Stage-specific metabolic reprogramming is also tightly coupled to functional specialization in the murine placenta, confirming the evolutionary conservation of this developmental principle [9].

#### 3.1.3. Hypoxia-Driven Metabolic Adaptation and Lactate Signaling

In the first-trimester human placenta, a physiological hypoxic microenvironment prevails [117]. HIF-1α and HIF-2α govern trophoblast stemness, cell fate and metabolic reprogramming [117,118]. Concomitantly, robust expression of HIFs, MCT1/4 and core glycolytic enzymes (HK2, PKM2, LDHA) establishes an acidic milieu at the maternal–fetal interface [17,119,120]. LDHA-derived lactate has been shown to stabilize trophoblast cell cycle progression via the PI3K/AKT/FOXO1/CyclinD1 pathway and suppress caspase-7-mediated apoptosis [121].

Beyond its role as a metabolic end product, lactate acts as a pleiotropic signaling molecule [17]. It signals through G protein-coupled receptor 81 and drives histone lactylation to regulate trophoblast and surrounding cell function [17,61]. In the human placenta, lactate is produced mainly by CTBs and EVTs, then imported into STBs via MCT1. Within STBs, lactate activates the PI3K/AKT/mTOR cascade to promote sterol regulatory element-binding protein 1-mediated transcription of stearoyl-CoA desaturase 1 and GPX4, thereby fortifying STBs against ferroptosis [122].

In RPL, the maternal–fetal interface displays a perturbed hypoxic niche, with bidirectional glycolytic dysfunction in trophoblasts [17,123]. In the hyperactive phenotype, excessive hypoxia upregulates LDHA, MCT-1, and MCT-4. The resulting lactate surplus is shuttled to dMφ, inducing pro-inflammatory M1 polarization and disrupting immune tolerance [120]. IL-33 deficiency exacerbates trophoblast senescence, further boosting glycolysis and lactate output. Lactate-dependent synaptosome-associated protein 29 lactylation triggers its degradation, impairing autophagy and mitochondrial function. Senotherapies, such as metformin and dasatinib, plus quercetin, target this senescence-linked metabolic dysregulation and improve pregnancy outcomes in mice [124]. In the defective phenotype, insufficient HIF-1α downregulates LDHA, restricts glycolysis, and suppresses PI3K/AKT/mTOR signaling. This impairs CTB and EVT proliferation and invasion while triggering lipid peroxidation and ferroptosis in STBs [109,121,122]. Diminished lactate compromises endothelial and macrophage function, aggravating placental damage and maternal–fetal crosstalk disruption [119,125]. Exogenous lactate miscarriage in LPS-induced mouse models [119,122] underscoring that balanced lactate signaling is critical for pregnancy maintenance.

#### 3.1.4. TCA Cycle Intermediates as Signaling Molecules

First-trimester trophoblasts predominantly rely on glycolysis, while succinate and itaconate, two TCA cycle metabolites, serve as key signaling molecules that distinctly regulate placental homeostasis and are implicated in RPL [109,126]. Succinate accumulates during early pregnancy, inhibiting prolyl hydroxylase domain proteins to stabilize HIF-1α [109], enhancing glycolysis and upregulating matrix metalloproteinases (MMPs) and vascular endothelial growth factor (VEGF) to facilitate trophoblast invasion and vascular remodeling [127]. Secreted succinate activates succinate receptor 1 (SUCNR1) on dMφ to promote M2 polarization [127,128]. In RPL, SDHB promoter hypomethylation drives SDHB overexpression, depleting succinate and blunting HIF-1α stabilization, thereby impairing trophoblast invasion and disrupting macrophage polarization [109,127,129]. Of note, aspirin reinstates succinate levels via SDH inhibition, mitigating pregnancy loss in mouse models [109].

Itaconate, produced by aconitate decarboxylase 1 (ACOD1), alkylates cysteine thiols to modify protein function [130]. In RPL villous tissues, elevated ACOD1 drives excessive itaconate production, which alkylates PI3K upstream regulators and suppresses the PI3K/AKT/folate receptor 1 (FOLR1) axis, impairing trophoblast invasion. Simultaneously, itaconate promotes M1 macrophage polarization, generating a pro-inflammatory microenvironment [130,131]. FOLR1 serves dual roles as a cell-surface folate transporter and a nuclear transcription factor that facilitates trophoblast invasion, migration, and EMT [132]. By inhibiting PI3K/Akt-FOLR1 pathway, itaconate links energy metabolism to folate-nucleotide metabolism dysfunction and serves as a metabolic-immune crosstalk hub RPL.

#### 3.1.5. HBP Pathway and O-Glcnacylation

The HBP acts as a glycolytic nutrient-sensing pathway, governing trophoblast function through O-GlcNAcylation [133,134]. Physiologically, mTOR preserves O-GlcNAc homeostasis via OGT modulation [135]. O-GlcNAcylation targets human chorionic gonadotropin, MMPs, GLUT1 and autophagy-related proteins to regulate trophoblast proliferation, invasion, differentiation and immune activity [49,133,136]. Hyperglycemia-induced HBP hyperactivation elevates O-GlcNAcylation, impairing trophoblast activity, provoking inflammatory and vascular lesions, and raising miscarriage risk [49,137,138]. Hypoxia elevates global O-GlcNAcylation, which activates GATA-binding protein 3-driven androgen overproduction and destabilizes HIF-1α, disrupting hypoxic adaptation and placental angiogenesis [137,139]. Though understudied in RPL, aberrant HBP-mediated O-GlcNAc homeostasis compromises trophoblast bioactivity and fetomaternal immune tolerance, serving as a potential pathological basis for RPL.

### 3.2. Lipid Metabolic Reprogramming

#### 3.2.1. Cholesterol Metabolism

EVTs exhibit a unique cholesterol metabolic profile characterized by enhanced expression of the high-density lipoprotein receptor scavenger receptor class B type I and diminished liver X receptor transcriptional activity [140]. This configuration maintains abundant cholesterol reserves for P4 synthesis, which is further supported by high hydroxy-delta-5-steroid dehydrogenase 3 beta- and steroid delta-isomerase 1 (HSD3B1) expression. In RPL, HSD3B1 is markedly reduced in EVTs, implicating progesterone deficiency in early pregnancy loss [140].

#### 3.2.2. Fatty Acid Metabolism

STBs derive their primary energy from FAO, which simultaneously steroidogenesis while sparing glucose for fetal allocation [15,21]. In RPL, villous total n-3 PUFAs and linoleic acid (C18:2n-6) are significantly reduced, accompanied by an elevated ARA (C20:4n 6) to eicosapentaenoic acid (C20:5n-3) ratio, indicating hyperactivation of the n-6 pathway and deficient production of n-3-derived anti-inflammatory mediators [63]. This imbalance indicates hyperactivation of the n-6 pathway and deficient production of n-3-derived anti-inflammatory mediators, skewing eicosanoid metabolism toward a pro-inflammatory profile [141,142]. Additionally, lipoxin A4 and resolvin D1 are specialized pro-resolving mediators (SPMs) derived from essential fatty acids that orchestrate inflammation resolution and promote trophoblast survival [143,144]. SPM insufficiency correlates with recurrent miscarriage, and aspirin or docosahexaenoic acid supplementation represents promising therapeutic interventions [143].

#### 3.2.3. Phospholipid Metabolism

Phospholipid metabolism provides structural components for membranes and generates signaling molecules that govern cell fate [145]. STB-derived microvesicles (STBMs), rich in sphingomyelin, cholesterol, and phospholipids, mediate maternal–fetal communication. In RPL, STBMs exhibit aberrant phospholipid remodeling, with elevated phosphatidylserine and reduced phosphatidylglycerol, PA, and ganglioside mannoside 3 that correlate with apoptosis, inflammation, and oxidative stress, contributing to trophoblast injury [146,147,148].

In infection-associated miscarriage, LPS-induced PLA2 and COX-2 upregulation enhances ARA conversion to PGE_2_ and leukotriene B_4_, while lipid peroxide accumulation drives an inflammatory-oxidative cycle in rat models [149]. N-acetylcysteine attenuates this by replenishing cysteine and strengthening antioxidant defenses [149]. In noninfectious URPL patients, disrupted COX-2/prostacyclin/peroxisome proliferator-activated receptor β/VEGFA signaling underscores the distinct involvement of phospholipid networks in angiogenesis and implantation [150]. Modulating critical phospholipid hubs may therefore advance individualized RPL treatment.

#### 3.2.4. Lipid Peroxidation and Ferroptosis

Ferroptosis, a lipid peroxidation-driven regulated cell death, contributes to RPL-associated trophoblast injury [111,151]. In human trophoblasts, membrane phospholipid-esterified PUFAs undergo peroxidation to generate toxic lipid peroxides such as malondialdehyde [151], while phospholipase PLA2G6 hydrolyzes peroxidized phosphatidylethanolamine to counteract ferroptosis induced by GPX4 inhibition or hypoxia–reoxygenation damage [152]. In RPL villi, untargeted metabolomics and transcriptomics reveal elevated lipid peroxidation, reduced cysteine, glutamate, and glutamine levels, and dysregulated expression of ferroptosis-related genes, including upregulated ACSL4 and downregulated GPX4 and solute carrier family 7 member 11 (SLC7A11), collectively leading to lipid peroxide buildup and trophoblast death [111]. Overall, phospholipid and fatty acid metabolic dysregulation synergistically promotes RPL through ferroptosis. Therapeutic strategies targeting ferroptosis through GPX4 enhancement, GSH supplementation, or iron metabolism modulation may improve pregnancy outcomes [151].

### 3.3. Amino Acid Metabolism

#### 3.3.1. Amino Acid Transport and mTOR Regulation

Human trophoblasts rely on a coordinated amino acid transport network to sustain proliferation, invasion, and fetal nutrient delivery [153]. System A (SNAT1/2/4) actively imports neutral non-essential amino acids, establishing a gradient that drives essential amino acid uptake via System L (LAT1/2 heterodimerized with 4F2hc) [154]. The cystine-glutamate exchanger SLC7A11 supports GSH synthesis, while SLC6A6 mediates taurine uptake [111,155]. CTBs upregulate System A/L to fuel proliferation and invasion, whereas STBs polarize these transporters to maximize fetal amino acid delivery [153,156].

This transporter network is centrally governed by the mTOR, which integrates amino acid availability with insulin and insulin-like growth factor 1 signaling [157]. mTOR controls SNAT2 and LAT1 membrane trafficking and activity, and tunes mitochondrial OXPHOS to energize substrate transport [158]. mTOR inhibition therefore disrupts transporter trafficking and autophagic flux, causing trophoblast proliferative arrest and defective invasion that elevate RPL risk [159,160]. In RPL trophoblasts, elevated LAT1 drives excess methionine uptake that disrupts one-carbon metabolism and induces global DNA hypermethylation [161], whereas diminished SLC7A11 impairs cystine import and GSH synthesis, sensitizing cells to ferroptosis [111]. Together, mTOR-dependent dysregulation of the amino acid transporter network contributes to RPL pathogenesis [116,153,160,162].

#### 3.3.2. Tryptophan Metabolism

Optimal tryptophan availability is indispensable for placental growth and fetal development [163,164]. IDO1-mediated tryptophan depletion restricts T-cell proliferation, while IDO1-activated signal transducer and activator of transcription 3 (STAT3) signaling upregulates MMP9, VEGFA and EMT-related genes [165,166,167]. The kynurenine pathway serves as the only de novo nicotinamide adenine dinucleotide (NAD^+^) synthetic route and sustains trophoblast redox homeostasis [168,169]. Tryptophan metabolism undergoes bidirectional dysregulation in RPL trophoblasts, mirroring disease heterogeneity. Reduced IDO1 expression disrupts maternal-fetal immune tolerance and trophoblast function [164,170]. Conversely, in a subset of RPL patients, interferon-γ strongly induces placental IDO1, triggering the kynurenine-AHR cascade that restricts trophoblast migration and invasion. Concurrently, interferon-γ stabilizes HIF-1α/aryl hydrocarbon receptor nuclear translocator, driving glycolytic reprogramming with TCA cycle suppression. Excessive kynurenine and lactate further deteriorate immune homeostasis and trophoblast performance, establishing a feedforward immunometabolic loop [171]. Physiological hypoxia suppresses IDO1, whereas excessive interferon-γ signaling overrides this restraint, and such IDO1 overexpression represents a maladaptive response that compromises trophoblast invasion and metabolic homeostasis [171]. L-tryptophan supplementation alleviates IDO1 deficiency-related trophoblast damage and immune rejection, while targeting the kynurenine-NAD^+^ axis offers an alternative strategy for RPL intervention [166,169].

#### 3.3.3. Arginine Metabolism

Arginine regulates trophoblast function through polyamines and NO production and its availability correlates with invasive capacity [172,173,174]. In ovine trophectoderm cells, basal proliferation persists even after complete blockade of polyamine and NO synthesis, suggesting that arginine also acts through metabolite-independent mechanisms [96,174]. Arginine metabolism exerts a Goldilocks effect. In placenta accreta spectrum disorders, excessive arginine hyperactivates the G protein coupled receptor family C group 6 member A/PI3K/AKT/MMP pathway, driving uncontrolled invasion [172], whereas in RPL this pathway is frequently underactivated, resulting in impaired invasion [113,125,172]. Restoring arginine availability or NO production may therefore improve pregnancy outcomes in RPL [173,174,175].

#### 3.3.4. Glutamine Metabolism

Glutamine metabolism sustains trophoblast bioenergetics and redox homeostasis through GLS1-mediated mitochondrial glutaminolysis, which fuels the TCA cycle and GSH synthesis [176,177]. Glutamine and its metabolite glutamate also act as signaling regulators of cellular bioenergetics, with L-glutamate identified as a key driver of murine placental metabolic switching [9,176]. In RPL, diminished villous GS and GLS1 expression, together with reduced glutamine and glutamate levels, are observed. Exogenous glutamine boosts trophoblast invasion by activating PI3K/AKT and upregulating collagen type I alpha 1 chain [178]. Concomitantly, RPL placentas exhibit GPX4 downregulation, GSH depletion, and lipid peroxide buildup, indicating convergent glutamine metabolic disturbance and oxidative stress that compromise trophoblast function [111].

Complementarily, levels of methionine, cysteine, and S-adenosylmethionine (SAM) are markedly reduced in RPL placentas [111]. SAM couples the folate-mediated one-carbon metabolism network with nucleotide biosynthesis [168,179]. Given the integrated roles of the methionine cycle, folate metabolism, and nucleotide synthesis in trophoblast epigenetic regulation and proliferation–differentiation dynamics, the following section focuses on one-carbon metabolism and its pathological remodeling in RPL.

### 3.4. One-Carbon Metabolism

One-carbon metabolism orchestrates folate-carbon unit flux into the methionine cycle and nucleotide biosynthesis, generating methyl donors and nucleic acid precursors that sustain trophoblast proliferation, differentiation, and epigenetic regulation.

#### 3.4.1. Folate-Mediated One-Carbon Metabolism

In human trophoblasts, folate is imported by the proton-coupled folate transporter, reduced folate carrier, and folate receptor alpha, and is then converted to tetrahydrofolate to enter a cooperative mitochondrial-cytosolic one-carbon network [179,180]. Mitochondrial one-carbon metabolism, initiated by serine hydroxymethyltransferase 2 (SHMT2) and methylenetetrahydrofolate dehydrogenase 2 (MTHFD2), generates 10-formyl-THF for purine biosynthesis. Meanwhile, cytosolic one-carbon metabolism, driven by serine hydroxymethyltransferase 1 (SHMT1), preferentially fuels pyrimidine synthesis and methyl donor production via methylenetetrahydrofolate reductase (MTHFR) [179,181]. These two compartments are coordinated through serine-glycine shuttling and formate transport [179,182]. mTOR signaling enhances folate uptake and couples to placental metabolic remodeling, and its disruption by folate deficiency impairs protein synthesis and mitochondrial respiration [180,183].

#### 3.4.2. Methionine Cycle

The methionine cycle serves as the execution hub for methylation reactions downstream of one-carbon metabolism [184]. Methionine synthase (MTR) transfers the methyl group from 5-methyl-THF to homocysteine (Hcy), generating methionine, which is converted to SAM, the universal methyl donor [185]. Following methyl transfer, SAM is hydrolyzed to S-adenosylhomocysteine (SAH) and then to Hcy, which can be remethylated to methionine or catabolized to cysteine [186]. In RPL placentas, reduced serine, methionine, cysteine, and SAM levels, together with elevated Hcy, indicate disruption of one-carbon metabolism in the trophoblasts [111,184,187]. Consistently, hyperhomocysteinemia has been shown to compromise trophoblast integrity through lysosomal destabilization, impaired autophagic flux, findings that have been validated in placental samples from spontaneous abortion patients with elevated Hcy [188,189].

#### 3.4.3. Nucleotide Metabolism and Adenosine Signaling

Trophoblast nucleotide biosynthesis employs de novo and salvage pathways coupled to proliferative and differentiation states [190]. In early gestation, CTBs and EVTs rely predominantly on de novo synthesis to sustain villous morphogenesis, whereas STBs shift toward the salvage pathway to minimize energy and substrate expenditure [182]. The mitochondrial one-carbon enzyme MTHFD2 sustains folate nucleotide flux to facilitate trophoblast DNA synthesis and EMT [181]. In RPL, MTHFD2 downregulation leads to 5-aminoimidazole-4-carboxamide ribonucleotide accumulation and AMP-activated protein kinase activation, with inactivation of the Janus kinase/STAT/Snail family transcriptional repressor 2 pathway, compromising trophoblast invasion and migration [181].

Nucleotides additionally serve as critical adenosine sources. In trophoblasts, the CD73/adenosine axis inhibits glycolysis via pyruvate dehydrogenase kinase 1 while adenosine-derived carbon supports the TCA cycle [182,191]. In URPL, suppressed transforming growth factor-β–mTOR–HIF-1α signaling reduces CD73 expression in EVTs, blunting ATP-to-adenosine conversion, restraining EVT proliferation and invasion, and contributing to maternal-fetal immune imbalance [192].

#### 3.4.4. Nicotinamide N-Methyltransferase (NNMT) at the Intersection of Methyl and NAD^+^ Metabolism

NNMT transfers a methyl group from SAM to nicotinamide (NAM), generating 1-methylnicotinamide and SAH. In RPL, NNMT downregulation leads to SAM overaccumulation and aberrant H3K27 methylation. This epigenetic modification suppresses cartilage oligomeric matrix protein (COMP) transcription, disrupts the COMP/CD36/ERK1/2 signaling cascade, and ultimately restricts trophoblast invasion [193]. Additionally, NNMT modulates NAD^+^ salvage synthesis by controlling cellular NAM bioavailability [9,169] and supplementation with NAD^+^ precursors could alleviate NAD^+^ deficiency related dysfunction and reduce the susceptibility to RPL [194,195,196].

### 3.5. Metabolic Network Integration in Trophoblasts

**Figure 6:**
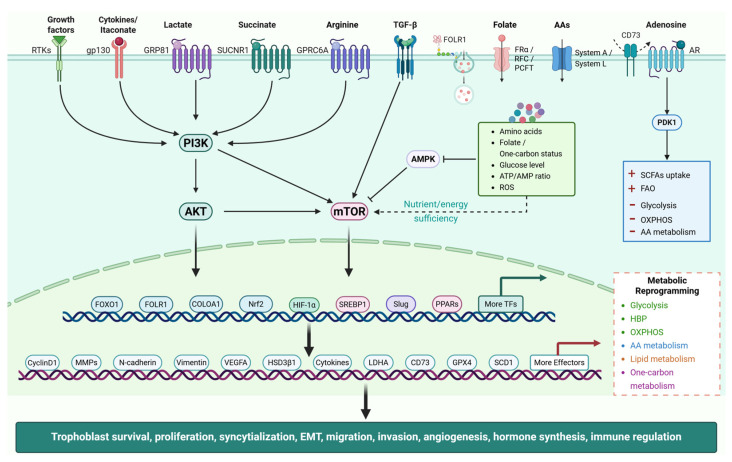
Key signaling networks governing metabolic reprogramming in trophoblasts. Metabolites and growth factors converge on PI3K/AKT and mTOR signaling hubs via their cognate receptors to orchestrate trophoblast metabolic rewiring. As a pivotal nutritional sensor, mTOR responds to cellular amino acid and folate availability, energy status, oxidative stress, and one-carbon metabolism to mediate biological functions. These pathways regulate transcription factors including FOXO1, HIF-1α, Nrf2, SREBP1, and PPARs, which cooperatively modulate glucose, lipid, amino acid and one-carbon metabolism. This integrative network underpins trophoblast survival, proliferation, syncytialization, EMT, migration, invasion, angiogenesis, hormone biosynthesis, and immune regulation, thereby safeguarding maternal-fetal metabolic homeostasis and orderly embryonic progression. Abbreviations: AMPK, adenosine monophosphate-activated protein kinase; AR, adenosine receptor; GPRC6A, G protein-coupled receptor class C group 6 member A; GRP81, G protein-coupled receptor 81; JAK, Janus kinase; Nrf2, nuclear factor erythroid 2-related factor 2; PCFT, proton-coupled folate transporter; PDK1, 3-phosphoinositide-dependent protein kinase 1; PPARs, peroxisome proliferator-activated receptors; RFC, reduced folate carrier; RTKs, receptor tyrosine kinases; SCD1, stearoyl-CoA desaturase 1; Slug, Snail family transcriptional repressor 2; SREBP1, sterol regulatory element-binding protein 1; TFs, transcription factors; gp130, glycoprotein 130. Created in BioRender. Runan, H. (2026) https://BioRender.com/hb8ojze (accessed on 8 July 2026).

**Figure 7:**
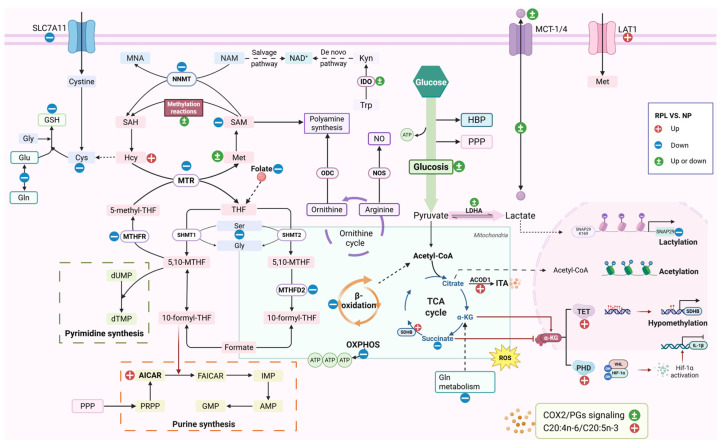
Aberrant metabolic reprogramming in trophoblasts from RPL. Compared with NP, trophoblasts from patients with RPL exhibit profound, network level metabolic dysregulation. Aberrant glycolysis induces dysregulated lactate signaling and histone lactylation. TCA cycle disruption is accompanied by altered levels of intermediate metabolites such as succinate and itaconate, driving inappropriate signal transduction. Notably, succinate accumulation inhibits α-KG-dependent TET and PHD activity, linking TCA cycle dysfunction to epigenetic and hypoxic signaling perturbations. FAO is attenuated, compromising energy supply. Defective Gln and Cys metabolism impair GSH synthesis, exacerbating mitochondrial damage. Concurrently, disturbances in phospholipid and fatty acid metabolism, together with dysregulated tryptophan, arginine, and BCAA metabolism, compromise trophoblast function and enhance pro-inflammatory signaling through distinct mechanisms that converge on similar functional deficits. Folate cycle blockade and disordered Met metabolism further impede nucleotide biosynthesis and related signaling. Collectively, the four core metabolic modules are tightly interconnected, and perturbation at any node can propagate through feed forward loops to destabilize the entire network, ultimately culminating in trophoblast dysfunction and pregnancy failure. Red plus symbols (+) indicate upregulation in RPL relative to NP, while blue minus symbols (−) indicate downregulation. Green plus-minus symbols (±) denote context dependent or bidirectional changes. Abbreviations: AICAR, 5-aminoimidazole-4-carboxamide ribonucleotide; AMP, adenosine monophosphate; FAICAR, formyl-5-aminoimidazole-4-carboxamide ribonucleotide; Gly, glycine; GMP, guanosine monophosphate; IMP, inosine monophosphate; ITA, itaconate; MNA, N-methylnicotinic acid; 5,10-MTHF, 5,10-methylenetetrahydrofolate; 10-formyl-THF, 10-formyltetrahydrofolate; 5-methyl-THF, 5-methyltetrahydrofolate; PHD, prolyl hydroxylase domain protein; PRPP, phosphoribosyl pyrophosphate; SNAP29, synaptosomal-associated protein 29; TET, ten-eleven translocation methylcytosine dioxygenase; THF, tetrahydrofolate; VHL, von Hippel-Lindau tumor suppressor; dTMP, deoxythymidine monophosphate; dUMP, deoxyuridine monophosphate. Created in BioRender. Runan, H. (2026) https://BioRender.com/q7orxgx (accessed on 8 July 2026).

Glucose, lipid, amino acid, and one-carbon metabolism constitute an integrated network in trophoblasts, coupled through shared intermediates and the PI3K/AKT/mTOR signaling hub [158] (Figure 6). In RPL, mTOR-dependent dysregulation produces bidirectional metabolic phenotypes. Glycolysis can be either deficient or excessive, TCA cycle metabolites can be depleted or accumulate, and amino acid pathways can be either suppressed or hyperactivated. These disturbances synergistically impair trophoblast proliferation, invasion, angiogenesis, and immunomodulation, and converge on ferroptosis as a defining metabolic cell death modality [111,121,125,130,197] (Figure 7). Metabolite-mediated cross-pathway communication reinforces metabolic coupling. Succinate stabilizes HIF-1α to promote lipid reprogramming [109,198], and itaconate disrupts folate-mediated one-carbon metabolism by inhibiting FOLR1 [130,132]. The methionine cycle supplies SAM for polyamine synthesis and phospholipid methylation [161,185], while NNMT links methyl and energy metabolism [199]. Fluctuations in SAM, acetyl-CoA, lactate, and succinate reshape DNA and histone modifications, establishing positive feedback loops that perpetuate metabolic reprogramming. This network-level integration means that disruption at any node can propagate system-wide, and combined strategies targeting mTOR, ferroptosis, NAD^+^ synthesis, and one-carbon metabolism may offer synergistic therapeutic avenues. The metabolic vulnerability of trophoblasts is further amplified by immune cell crosstalk and systemic metabolic disturbances, as discussed in Section 4.1 and Section 4.2.

## 4. Perspectives and Future Directions

### 4.1. Metabolic Regulation of Decidual Immune Homeostasis

The metabolic reprogramming of DSCs and trophoblasts extends beyond cell autonomous effects to orchestrate the function of decidual immune cells through a network of secreted metabolites [12,200]. In healthy pregnancy, DSC derived FBP drives IL-27 dependent M2 macrophage polarization [56], while IL-15 activates mTOR signaling in dNK cells to sustain a pro-angiogenic, low cytotoxicity phenotype [8]. Trophoblast derived lactate stabilizes M2 macrophages, and succinate signals through SUCNR1 to maintain antioxidant defenses in dMφ [109,126,128]. Multiple cell types cooperatively express IDO, generating physiological kynurenine levels that preserve immune tolerance and angiogenesis [101,102,166,171]. CD39 and CD73 on dNK cells and trophoblasts convert ATP to adenosine, suppressing local inflammation [192,201]. In RPL, this homeostatic network is disrupted [8,12,202]. Trophoblast metabolic dysregulation promotes M1 polarization via excess glycolytic lactate, surplus itaconate, and deficient TCA-derived succinate [109,120,130], while DSC derived arachidonic acid transfer via CD36 fuels COX-2 dependent inflammation in dMφ [8,202]. Pathological kynurenine accumulation in dMφ triggers AHR-dependent lysosomal dysfunction and mitochondrial damage, leading to mtDNA release and cyclic GMP-AMP synthase–stimulator of interferon genes–driven pro-inflammatory cytokine secretion [203]. These pro-inflammatory mediators reciprocally suppress FBP, succinate, and LPA synthesis in DSCs and trophoblasts, establishing a self-reinforcing inflammatory loop [3]. Concurrently, dNK cells shift from a pro-angiogenic dNK1 phenotype toward a cytotoxic dNK3 phenotype with elevated perforin and granzyme B, directly targeting trophoblasts [204,205]. This multicellular metabolic-inflammatory circuit represents a feedforward axis that perpetuates pregnancy loss, and its interruption may offer therapeutic opportunities.

### 4.2. Systemic-Local Metabolic Axes in RPL

Maternal systemic metabolic status, shaped by the gut microbiome, nutritional intake, pre-existing metabolic disorders, and environmental exposures, remotely influences the metabolic microenvironment of the maternal–fetal interface [108,206]. Gut microbiota-derived short-chain fatty acids and tryptophan metabolites promote peripheral regulatory T-cell expansion and restrain pathogenic T-cell responses at the interface, while microbial carbohydrate metabolites directly modulate dNK cell glycolytic and effector function [202,207]. Gut dysbiosis alone is sufficient to disrupt decidual immune homeostasis and precipitate fetal loss in mouse models, whereas dietary fiber or probiotic interventions restore protective metabolite production and immune tolerance [206,207,208]. Nutritional insufficiency further compromises local metabolism. Dietary folate and vitamin B12 deficiency synergizes with MTHFR and methionine synthase reductase (MTRR) loss-of-function variants to elevate circulating Hcy, which damages placental vascular endothelium and impairs trophoblast function [187,188,209,210]. Similarly, inadequate intake of tryptophan and vitamin B3 restricts NAD^+^ synthesis, and when combined with maternal 3-hydroxyanthranilate 3,4-dioxygenase or kynureninase loss-of-function variants or hypoxia, NAD deficiency impairs embryo development and markedly increases the risk of embryo loss [194,195,196]. Environmental endocrine disruptors, including bisphenol A, nanoplastics, and cigarette smoke, impair placental glycolysis, lipid metabolism, and tryptophan catabolism, triggering trophoblast ferroptosis and hindering vascular remodeling and decidual immune tolerance [211,212,213,214]. Maternal obesity and hyperglycemia drive HBP hyperactivation and excessive O-GlcNAcylation in trophoblasts [49,133,139], while hyperinsulinemia paradoxically suppresses endometrial GLUT1, limiting glucose availability for decidualization [29,30,35,108]. Collectively, these systemic inputs converge on the same metabolic pathways dysregulated locally in DSCs and trophoblasts, suggesting that RPL risk reflects an integrated gene–diet–microbiome–environment–metabolism axis. Longitudinal studies integrating preconception metabolomic, microbiome, and genetic profiling will be essential to identify high risk women and to test whether systemic metabolic correction before pregnancy can reduce RPL incidence.

### 4.3. Translational Implications

The metabolic alterations cataloged in this review offer a foundation for developing diagnostic biomarkers and therapeutic strategies for RPL. Among candidate non-invasive biomarkers, serum homocysteine is consistently elevated in RPL cohorts and reflects the combined effect of dietary folate deficiency and *MTHFR*/*MTR*/*MTRR* genetic variants on one-carbon flux [187,209,215,216]. The plasma kynurenine-to-tryptophan ratio reflects decidual IDO1 activity and may identify women with impaired maternal–fetal immune tolerance [102,164], while STBMs carry a placenta-specific lipid signature detectable in maternal circulation [148]. Decidual or circulating myeloid-derived suppressor cells, RORγt^+^ regulatory T-cells, and tryptophan metabolite profiles provide additional tools for etiological classification [170,207].

From a therapeutic perspective, several metabolite-based interventions have demonstrated proof of concept in preclinical models. Metabolic interventions, including supplementation of FBP, succinate, TMAO, α-KG, and NAD^+^ precursors, restore decidualization and placentation [56,92,93,109,195], while ferroptosis inhibitors, such as ferrostatin-1, cysteine, and GSH, attenuate trophoblast and DSC death [94,111,151]. Aspirin restores succinate levels via SDH inhibition [109], and metformin protects against DSC and trophoblast senescence [106,124]. Gut-immune axis restoration through dietary tryptophan, AHR agonists, or tryptophan-metabolizing probiotics reduces fetal resorption in preclinical models, while antibiotics that deplete indole-producing commensals, particularly vancomycin, should be used cautiously during pregnancy [202,207]. Certain traditional Chinese medicine formulations and bioactive compounds, including Shoutai Wan and baicalein, have shown preclinical efficacy in reducing pregnancy loss [119,170,217,218,219]. Given the bidirectional metabolic dysfunction and multicellular crosstalk that characterize RPL, synergistic targeting of multiple metabolic nodes may achieve superior outcomes compared with single-pathway blockade. The major metabolic alterations, their associated molecular pathways, and their therapeutic implications are summarized in Table 1.

## 5. Conclusions

This review has synthesized current evidence establishing metabolic reprogramming at the maternal–fetal interface as a central driver of RPL. Disturbed glucose, lipid, amino acid, redox, and one-carbon metabolism in DSCs and trophoblasts triggers a cascade of pathological consequences, ranging from impaired decidualization and vascular defects to immune imbalance and failed placentation, which collectively underpin RPL development. These cell-intrinsic metabolic defects are further amplified by disrupted metabolic crosstalk with decidual immune cells and by systemic inputs from the gut microbiome, nutritional status, and metabolic disorders, together collapsing maternal–fetal immune privilege.

Despite these advances, critical gaps remain. The temporal relationship between metabolic dysregulation and pregnancy failure is unresolved, as most human data derive from samples collected after miscarriage has occurred. The metabolic heterogeneity of RPL, exemplified by bidirectional glycolytic phenotypes in trophoblasts and divergent IDO1 activity patterns, demands patient stratification beyond single-marker classification. Furthermore, the long-term metabolic consequences for surviving offspring remain largely unexplored.

Emerging technologies, including spatial multi-omics, single-cell metabolomics, and trophoblast-decidual organoid models, are poised to resolve the spatiotemporal dynamics of metabolic reprogramming at unprecedented resolution. Rigorous prospective cohort studies and subtype-stratified clinical trials will be essential to translate these mechanistic insights into validated biomarkers and targeted therapies. Ultimately, a systems-level understanding that integrates local cellular metabolism, intercellular crosstalk, and systemic metabolic influences will be required to advance precision medicine for RPL.

## Figures and Tables

**Table 1:** Major metabolic alterations in DSCs and trophoblasts associated with RPL.

Cell Type	Metabolic Pathway	Key Alterations in RPL	Molecular Pathway	Evidence	Biological Consequence	Therapeutic Potential (Ref.)
DSCs	Glycolysis/histone lactylation	HK2 ↑, lactate ↑, aberrant H3K18la at CUX1 promoter, SASP activation	HK2–lactate–H3K18la–CUX1–SASP	Human, Mouse	DSC senescence, decidualization failure	CUX1 depletion or glycolysis inhibition [55]
DSCs	PPP	GPR120 ↓, GLUT1/G6PD-mediated PPP flux ↓	GPR120–ERK/AMPK–FOXO1–GLUT1–PPP	Human, Mouse	Impaired glucose uptake, decidualization failure	ω-3 PUFA (GPR120 agonist) [25]
DSCs	Glycolysis/immunometabolism	FBP ↓, IL-27 ↓, M2 macrophage ↓	FBP–PKM2–IL-27–COX-2^+^ M2 macrophage	Human, Mouse	Impaired maternal–fetal immune tolerance	FBP supplementation [56]
DSCs	Glycolysis (DSC subtypes)	glyDSCs and iDSCs enriched, HIF-1α/GLUT1/HK2/LDHA ↑	HIF-1α–glycolysis–inflammation loop	Human	Decidual inflammation, immune dysregulation	Combination therapy targeting DSCs [14]
DSCs	Glucose uptake/glycogen storage	SGLT1 ↓	SGLT1–glucose uptake	Human	Decidualization failure, implantation failure	Caution with SGLT1 inhibitors [38]
DSCs	FAO	CPT1A ↓, free carnitine and acylcarnitines ↓	CPT1A–FAO–PI3K/AKT/FOXO1	Human, Mouse	DSC energy depletion, decidualization failure	Carnitine supplementation (metabolomic evidence) [19,73]
DSCs	Glycerophospholipid metabolism	PC, PE, PS, PG, LPC, LPE ↓, PLA2G2E ↓	PLA2–glycerophospholipid remodeling	Human	Membrane remodeling disrupted, eicosanoid synthesis impaired	[19,63]
DSCs	Essential fatty acid metabolism	Total n-3 PUFA ↓	n-3 PUFA–immune regulation	Human	Impaired immune modulation, vascular remodeling	n-3 PUFA supplementation [63]
DSCs	Sphingolipid/NETosis	SphK deficiency, excessive NET formation	SphK–S1P–PAD4–NETs	Mouse, Human cell	Decidual vascular damage, immune thrombosis	PAD4 inhibitor [91]
DSCs	Sphingolipid/SM accumulation	SGMS1 ↑, SM subtype accumulation	SGMS1–SM	Human	Decidual dysfunction	[19]
DSCs	ARA metabolism	Excess lipid transfer to dMφ via CD36, COX-2/PGE_2_/IL-1β ↑,ARA ↑	ARA–CD36–COX-2–PGE_2_/IL-1β	Human, Mouse	Decidual inflammation, immune imbalance	PRL supplementation [83,85]
DSCs	Glutamine metabolism	Glutaminolysis impaired, α-KG ↓	Gln–Glu–α-KG–histone demethylation/ATP	Human, Mouse	Epigenetic dysregulation, energy deficit	α-KG supplementation [93]
DSCs	Iron/ferroptosis	Ferroportin ↓, iron retention, GSH/GPX4 ↓, lipid ROS ↑	Ferroportin–iron–GSH/GPX4–lipid ROS	Human, Mouse	DSC ferroptosis, decidual dysfunction	Ferroptosis inhibitor/GSH or cysteine [94]
DSCs	BCAA metabolism	SLC3A2 ↑, leucine uptake ↑, p38 MAPK activation	SLC3A2–leucine–p38 MAPK	Human, Mouse	DSC senescence	Leucine restriction or metformin [106]
DSCs	TMAO/FMO3	FMO3 ↓, TMAO ↓, 14-3-3η–PDK1 interaction impaired	FMO3–TMAO–14-3-3η–PDK1–FOXO1	Human, Mouse	FOXO1 nuclear translocation blocked	TMAO supplementation [92]
DSCs	Arginine metabolism	L-citrulline ↑, arginine-to-NO conversion defective	ASS1–arginine–NO	Human	Impaired decidual perfusion, immune dysregulation	[19]
Trophoblasts	Glucose transport	SLC2A1 (GLUT1) ↓	GLUT1–PI3K/AKT–EMT	Human cell	Mitochondrial stress, invasion impaired	Restore GLUT1 [115,116]
Trophoblasts	Glycolysis/lactate (defective)	LDHA ↓, lactate ↓, PI3K/AKT/mTOR ↓	HIF-1α–LDHA–lactate–PI3K/AKT/mTOR	Human, Cell	Cell cycle arrest, apoptosis, invasion ↓	Restore LDHA or lactate [121]
Trophoblasts	Glycolysis/lactate (hyperactive)	LDHA/MCT-1/MCT-4 ↑, lactate surplus, M1 polarization	HIF-1α–LDHA–lactate–MCT1/4–M1	Human, Mouse	Immune intolerance, inflammation	MCT-1 inhibitor AZD3965 [120]
Trophoblasts	Lactate/ferroptosis resistance	CTB–STB lactate shuttle impaired, SCD1/GPX4 ↓	MCT1–lactate–PI3K/AKT–SCD1/GPX4	Human, Mouse	STB ferroptosis, placental dysfunction	Lactate supplementation [122]
Trophoblasts	Glycolysis/lactate–SNAP29 lactylation	Senescent trophoblasts: glycolysis ↑, lactate ↑, SNAP29 K169 lactylation, autophagy impairment	IL-33–lactate–SNAP29 lactylation–autophagy–senescence	Human, Mouse	Trophoblast senescence, invasion ↓	Metformin/dasatinib + quercetin [124]
Trophoblasts	TCA/succinate	SDHB hypomethylation, succinate ↓, HIF-1α blunted	SDHB–succinate–PHD2–HIF-1α	Human, Mouse	EVT invasion ↓, macrophage polarization disrupted	Succinate/aspirin [109]
Trophoblasts	TCA/itaconate	ACOD1 ↑, itaconate ↑, PI3K/AKT suppressed	ACOD1–itaconate–PI3K/AKT–FOLR1	Human, Mouse	Invasion ↓, M1 polarization, folate disruption	ACOD1 inhibition [130]
Trophoblasts	Cholesterol/progesterone	HSD3B1 ↓, local progesterone synthesis impaired	SR-BI–LXR–HSD3B1–progesterone	Human	Immune dysregulation, receptivity ↓	Progesterone [140]
Trophoblasts	Fatty acid/SPMs	n-3 PUFAs ↓, n-6/n-3 ↑, SPMs deficient	PLA2–COX–LOX–SPMs	Human	Pro-inflammatory eicosanoid shift	Aspirin/docosahexaenoic acid [63,143,144]
Trophoblasts	ARA/COX-2–PGI_2_–VEGF	COX-2 ↓, PGF_2_α ↓, PGD_2_ ↓, PGE_2_ ↓, PGI_2_ ↓, VEGF-A ↓	COX-2/PGI_2_/PPARβ/RXRα/VEGF-A	Human	Impaired angiogenesis and invasion	Restore COX-2/PGI_2_ signaling [150]
Trophoblasts	Glycerophospholipid/sphingolipid (STBM)	PI ↓, PA ↓, GM3 ↓, PS ↑ in STBM	STBM lipid remodeling	Human (preliminary)	Altered immune regulation, coagulation, oxidative stress	Non-invasive liquid biopsy potential; validation needed [148]
Trophoblasts	Phospholipid/oxidative stress	cPLA_2_ ↑, sPLA_2_ ↑, COX-2 ↑, PGE_2_ ↑, LTB_4_ ↑, lipid peroxidation ↑	PLA_2_–COX-2–PGE_2_/LTB_4_–oxidative stress	Rat	Placental inflammation, oxidative damage, fetal death	N-acetylcysteine (antioxidant) [149]
Trophoblasts	Phospholipid/ferroptosis	GPX4/SLC7A11 ↓, ACSL4 ↑, GSH ↓, lipid peroxide ↑, lipid ROS ↑	SLC7A11–GSH–GPX4–ACSL4–lipid ROS	Human, Mouse	Trophoblast ferroptosis	GPX4 enhancer/GSH/Fer-1 [111,151]/Modified Shoutai Pill [218]/Baicalein [217]
Trophoblasts	AAT/mTOR (LAT1)	LAT1 ↑, methionine uptake ↑, DNA hypermethylation	mTOR–LAT1–methionine–SAM–DNA methylation	Human, Mouse	Epigenetic dysregulation	Target LAT1 [161]
Trophoblasts	AAT/ferroptosis (SLC7A11)	SLC7A11 ↓, cystine import ↓, GSH ↓	mTOR–SLC7A11–GSH–GPX4	Human	Redox imbalance, ferroptosis	Restore SLC7A11 [111]
Trophoblasts	Glutamine/PI3K/AKT/COL1A1	Gln ↓, GS ↓, GLS ↓	Gln–PI3K/AKT–COL1A1	Human, Cell	Impaired invasion and proliferation	Glutamine supplementation [178]
Trophoblasts	Tryptophan (IDO1 deficient)	IDO1 ↓, Kyn ↓, VEGFA/EMT ↓	IDO1–Kyn–VEGFA/EMT	Human, Cell	Invasion ↓, vascular remodeling ↓	Restore IDO1 or Kyn [164,166]
Trophoblasts	Tryptophan (IDO1 hyperactive)	IFN-γ–IDO1 ↑, Kyn–AHR cascade, glycolysis ↑	IFN-γ–IDO1–Kyn–AHR–HIF-1α–glycolysis	Human, Cell	Invasion restricted, immune dysregulation	Target IFN-γ/IDO1 [171]
Trophoblasts	Folate one-carbon	MTHFD2 ↓, AICAR ↑, AMPK ↑, JAK/STAT/Slug ↓	MTHFD2–AICAR–AMPK–JAK/STAT/Slug	Human, Mouse, Cell	Nucleotide synthesis ↓, EMT ↓	Restore MTHFD2 [181]
Trophoblasts	Methionine cycle	Serine/methionine/cysteine/SAM ↓, Hcy ↑	MTR–MTHFR–SAM–SAH–Hcy	Human, Mouse, Cell	Impaired placental development	Active folate/B12 [168,188,189]
Trophoblasts	Nicotinamide/NNMT	NNMT ↓, SAM ↑, H3K27 hypermethylation, COMP ↓	NNMT–SAM–H3K27 hypermethylation–COMP–CD36–ERK1/2	Human, Mouse, Cell	Invasion ↓	Restore NNMT or COMP [193]
Trophoblasts	NAD^+^ synthesis	HAAO/KYNU loss or precursor deficiency, NAD^+^ ↓	HAAO–KYNU–NAD^+^	Human, Mouse	Multi-pathway metabolic disruption, embryo loss	Tryptophan/vitamin B3 [195]
Trophoblasts	Purinergic/adenosine	CD73 ↓, adenosine ↓, A2aR signaling ↓	CD73–adenosine–A2aR–M1/M2	Human, Cell	dNK cytotoxicity ↑, M1 macrophage ↑, trophoblast invasion ↓	Adenosine receptor agonist [191,192,201]

Arrows: ↑ = increased level/function in RPL vs normal pregnancy; ↓ = decreased level/function. Abbreviations: ACOD1, aconitate decarboxylase 1; ACSL4, acyl-CoA synthetase long chain family member 4; AHR, aryl hydrocarbon receptor; AICAR, 5-aminoimidazole-4-carboxamide ribonucleotide; AKT, AKT serine/threonine kinase; AMPK, adenosine monophosphate-activated protein kinase; ARA, arachidonic acid; ASS1, argininosuccinate synthase 1; BCAA, branched-chain amino acid; CD36, cluster of differentiation 36; CD73, ecto-5’-nucleotidase; COL1A1, collagen type I alpha 1 chain; COMP, cartilage oligomeric matrix protein; COX-2, cyclooxygenase-2; CPT1A, carnitine palmitoyltransferase 1A; CTB, cytotrophoblast; CUX1, cut-like homeobox 1; DSCs, decidual stromal cells; dMφ, decidual macrophages; dNK, decidual natural killer cells; EMT, epithelial–mesenchymal transition; ERK, extracellular signal-regulated kinase; EVT, extravillous trophoblast; FAO, fatty acid oxidation; FBP, fructose-1,6-bisphosphate; FMO3, flavin-containing monooxygenase 3; FOLR1, folate receptor 1; FOXO1, forkhead box protein O1; G6PD, glucose-6-phosphate dehydrogenase; Gln, glutamine; GLS, glutaminase; Glu, glutamate; GLUT1, glucose transporter 1; GPR120, G protein-coupled receptor 120; GPX4, glutathione peroxidase 4; GS, glutamine synthetase; GSH, glutathione; H3K18la, histone H3 lysine 18 lactylation; HAAO, 3-hydroxyanthranilate 3,4-dioxygenase;Hcy, homocysteine; HIF-1α, hypoxia-inducible factor 1α; HK2, hexokinase 2; HSD3B1, hydroxy-delta-5-steroid dehydrogenase 3 beta- and steroid delta-isomerase 1; IDO1, indoleamine 2,3-dioxygenase 1; IFN-γ, interferon-γ; IL-1β, interleukin-1β; IL-27, interleukin-27; JAK, Janus kinase; KYNU, kynureninase; Kyn, kynurenine; LAT1, large neutral amino acid transporter 1; LDHA, lactate dehydrogenase A; LPC, lysophosphatidylcholine; LPE, lysophosphatidylethanolamine; LTB4, leukotriene B4; MCT1/4, monocarboxylate transporter 1/4;MTHFD2, methylenetetrahydrofolate dehydrogenase 2; MTHFR, methylenetetrahydrofolate reductase; MTR, methionine synthase; mTOR, mechanistic target of rapamycin; NAD^+^, nicotinamide adenine dinucleotide; NETs, neutrophil extracellular traps; NNMT, nicotinamide N-methyltransferase; NO, nitric oxide; PAD4, peptidyl arginine deiminase 4; PC, phosphatidylcholine; PDK1, pyruvate dehydrogenase kinase 1; PE, phosphatidylethanolamine; PG, phosphatidylglycerol; PGD2, prostaglandin D2; PGE2, prostaglandin E2; PGF2α, prostaglandin F2 alpha; PHD, prolyl hydroxylase domain; PI, phosphatidylinositol; PI3K, phosphatidylinositol 3-kinase; PKM2, pyruvate kinase M2; PLA2, phospholipase A2; PLA2G2E, phospholipase A2 group IIE; PPP, pentose phosphate pathway; PRL, prolactin; PS, phosphatidylserine; PUFA, polyunsaturated fatty acid; ROS, reactive oxygen species; RPL, recurrent pregnancy loss; SAM, S-adenosylmethionine; SASP, senescence-associated secretory phenotype; SCD1, stearoyl-CoA desaturase 1; SDHB, succinate dehydrogenase complex iron sulfur subunit B; SGMS1, sphingomyelin synthase 1; SGLT1, sodium–glucose-linked transporter 1; SLC2A1, solute carrier family 2 member 1; SLC3A2, solute carrier family 3 member 2; SLC7A11, solute carrier family 7 member 11; SM, sphingomyelin; SNAP29, synaptosome associated protein 29; SphK, sphingosine kinase; S1P, sphingosine-1-phosphate; SPM, specialized pro-resolving mediator; SR-BI, scavenger receptor class B type I; STAT, signal transducer and activator of transcription; STB, syncytiotrophoblast; STBM, syncytiotrophoblast-derived microvesicle; TCA, tricarboxylic acid cycle; TMAO, trimethylamine N-oxide; VEGF, vascular endothelial growth factor; VEGFA, vascular endothelial growth factor A; α-KG, alpha-ketoglutarate; ω-3 PUFA, omega-3 polyunsaturated fatty acid.

## Data Availability

No new data were created or analyzed in this study. Data sharing is not applicable to this article.

## Supplementary Materials

The following supporting information can be downloaded at https://www.mdpi.com/article/10.3390/ijms27146413/s1.

## Abbreviations

The following abbreviations are used in this manuscript:

ACOD1	Aconitate decarboxylase 1
ACSL1/4	Long-chain Acyl-CoA synthetase 1/4
AHR	Aryl hydrocarbon receptor
AKT	Protein kinase B
ARA	Arachidonic acid
ATX	Autotaxin
BCAA	Branched-chain amino acid
CD	Cluster of differentiation
COMP	Cartilage oligomeric matrix protein
COX	Cyclooxygenase
CPT1/2	Carnitine palmitoyltransferase 1 and 2
CREB	cAMP response element-binding protein
CTB	Cytotrophoblast
CUX1	Cut-like homeobox 1
dMφ	Decidual macrophages
dNK	Decidual natural killer
DSC	Decidual stromal cell
EMT	Epithelial–mesenchymal transition
EnSC	Endometrial stromal cell
EP2/EP4	Prostaglandin e receptor 2/prostaglandin e receptor 4
EVT	Extravillous trophoblast
ERK	Extracellular signal-regulated kinase
FAO	Fatty acid oxidation
FBP	Fructose-1,6-bisphosphate
FMO3	Flavin-containing monooxygenase 3
FOXO1	Forkhead box protein o1
FOLR1	Folate receptor 1
G6PD	Glucose-6-phosphate dehydrogenase
GLS1	Glutaminase 1
GLUT	Glucose transporter
GPX4	Glutathione peroxidase 4
GSH	Glutathione
GS	Glutamine synthetase
H3K18la	Histone H3 lysine 18 lactylation
H3K27	Histone H3 lysine 27
HBP	Hexosamine biosynthesis pathway
Hcy	Homocysteine
HIF1α/HIF2α	Hypoxia-inducible factor 1α/Hypoxia-inducible factor 2α
HK2	Hexokinase 2
HSD3B1	Hydroxy-delta-5-steroid dehydrogenase 3 beta- and steroid delta-isomerase 1
IDO1	Indoleamine 2,3-dioxygenase 1
IGFBP1	Insulin-like growth factor binding protein 1
IL	Interleukin
IRS2	Insulin receptor substrate 2
LAT1/2	L-type amino acid transporter 1/2
LDHA	Lactate dehydrogenase a
LPA	Lysophosphatidic acid
LPAR	Lysophosphatidic acid receptor
LPC	Lysophosphatidylcholine
LPP1/3	Lipid phosphate phosphatase 1/3
MAPK	Mitogen-activated protein kinase
MCT1/MCT4	Monocarboxylate transporter 1/monocarboxylate transporter 4
MMP	Matrix metalloproteinase
mTOR	Mechanistic target of rapamycin
MTHFD2	Methylenetetrahydrofolate dehydrogenase 2
MTHFR	Methylenetetrahydrofolate reductase
MTR	Methionine synthase
MTRR	Methionine synthase reductase
NNMT	Nicotinamide n-methyltransferase
NO	Nitric oxide
NOS	Nitric oxide synthase
ODC	Ornithine decarboxylase
OGT	O-Linked β-N-Acetylglucosamine Transferase
OXPHOS	Oxidative phosphorylation
PA	Phosphatidic acid
PDK1	3-phosphoinositide-dependent protein kinase 1
PFK1	Phosphofructokinase 1
PG	Prostaglandin
PI3K	Phosphatidylinositol 3-kinase
PKA	Protein kinase A
PKM2	Pyruvate kinase M2
PLA2	Phospholipase A2
PPP	Pentose phosphate pathway
PR	Progesterone receptor
PRL	Prolactin
PUFA	Polyunsaturated fatty acid
RPL	Recurrent pregnancy loss
SAM	S-adenosylmethionine
SAH	S-adenosylhomocysteine
scRNA-seq	Single-cell RNA sequencing
SDHB	Succinate dehydrogenase B
SGLT1	Sodium–glucose-linked transporter 1
SLC3A2	Solute carrier family 3 member 2
SLC7A11	Solute carrier family 7 member 11
S1P	Sphingosine-1-phosphate
SPHK1	Sphingosine kinase 1
SASP	Senescence-associated secretory phenotype
SHMT	Serine hydroxymethyltransferase
STB	Syncytiotrophoblast
STBM	Syncytiotrophoblast-derived microvesicles
SUCNR1	Succinate receptor 1
TCA	Tricarboxylic acid
TE	Trophectoderm
TMAO	Trimethylamine n-oxide
TSC	Trophoblast stem cell
UDP-GlcNAc	Uridine diphosphate N-acetylglucosamine
URPL	Unexplained recurrent pregnancy loss
VEGF	Vascular endothelial growth factor
YAP1	Yes-associated protein 1
α-KG	α-ketoglutarate
