# Supplementary material for: Metabolic Reprogramming at the Maternal–Fetal Interface: Insights from Decidual Stromal Cells and Trophoblasts in Healthy Pregnancy Versus Recurrent Pregnancy Loss
Source: Int J Mol Sci. 2026 Jul 19;27(14):6413. doi: 10.3390/ijms27146413 (PMC13409969; doi:10.3390/ijms27146413)
Supplement: Supplementary file 1 [file ijms-27-06413-s001.zip › ijms-4347396-supplementary.pdf]

# Supplementary Materials

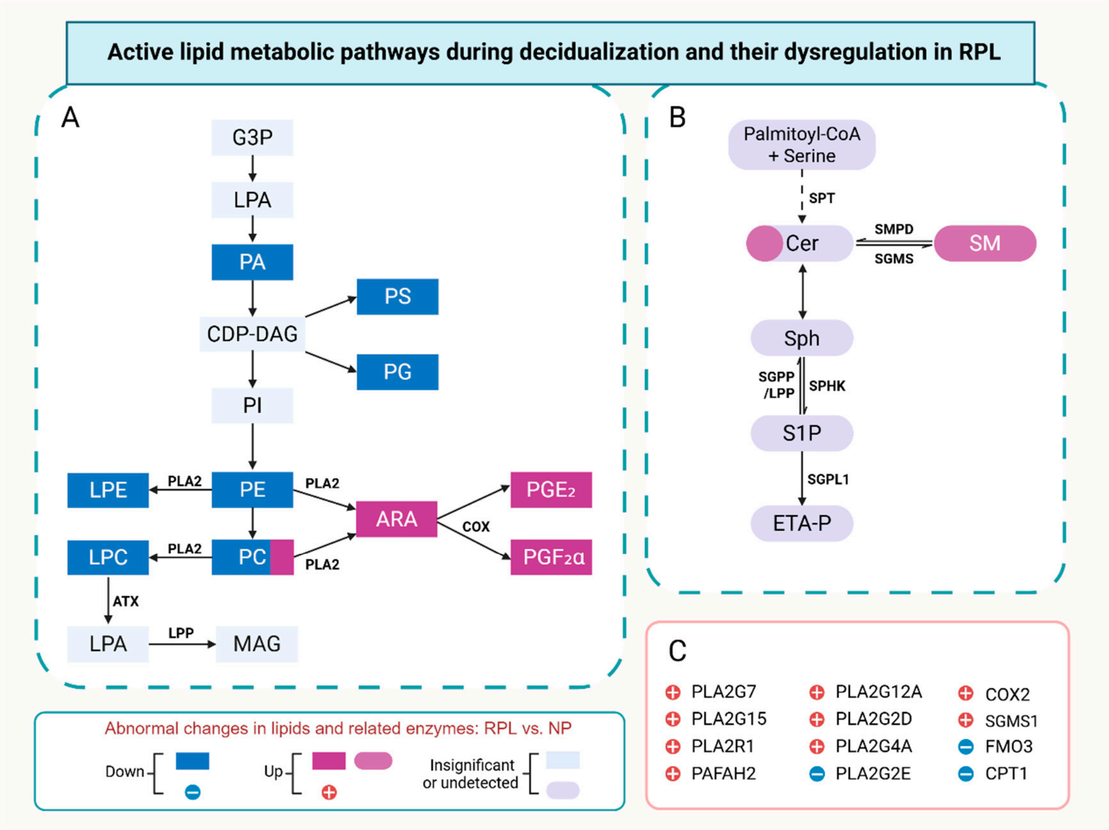


**Figure S1.** Remodeling of lipid metabolic pathways in healthy decidualization versus RPL. **(A)** Glycerophospholipid metabolic pathway. **(B)** Sphingolipid metabolic pathway. **(C)** Summary of altered lipids and corresponding enzymes in DSCs from RPL patients relative to NP. Fatty acid oxidation and related dysregulation are depicted in Figure 4. Abbreviations: ARA, arachidonic acid; ATX, autotaxin; CDP-DAG, cytidine diphosphate diacylglycerol; Cer, ceramide; COX, cyclooxygenase; COX2, cyclooxygenase 2; CPT1, carnitine palmitoyltransferase 1; ETA-P, ethanolamine phosphate; FMO3, flavin-containing monooxygenase 3; G3P, glycerol-3-phosphate; LPA, lysophosphatidic acid; LPC, lysophosphatidylcholine; LPE, lysophosphatidylethanolamine; LPP, lipid phosphate phosphatase; MAG, monoacylglycerol; NP, normal pregnancy; PA, phosphatidic acid; PAFAH2, platelet-activating factor acetylhydrolase 2; PC, phosphatidylcholine; PE, phosphatidylethanolamine; PG, phosphatidylglycerol; $PGE_2$, prostaglandin E2; $PGF_{2\alpha}$, prostaglandin F2$\alpha$; PI, phosphatidylinositol; PLA2, phospholipase A2; PLA2G7, phospholipase A2 group VII; PLA2G12A, phospholipase A2 group XIIA; PLA2G15, phospholipase A2 group XV; PLA2G2D, phospholipase A2 group IID; PLA2G2E, phospholipase A2 group IIE; PLA2G4A, phospholipase A2 group IVA; PLA2R1, phospholipase A2 receptor 1; RPL, recurrent pregnancy loss; S1P, sphingosine-1-phosphate; SGMS, sphingomyelin synthase; SGMS1, sphingomyelin synthase 1; SGPP, sphingosine-1-phosphate phosphatase; SGPL1, sphingosine-1-phosphate lyase; SM, sphingomyelin; Sph, sphingosine; SPHK, sphingosine kinase; SMPD, sphingomyelin phosphodiesterase; SPT, serine palmitoyltransferase. Created in BioRender. Runan, H. (2026) https://BioRender.com/slf3fjl (accessed 8 July 2026).
